# Part II. Common questions and misconceptions about creatine supplementation: what does the scientific evidence really show?

**DOI:** 10.1080/15502783.2024.2441760

**Published:** 2024-12-25

**Authors:** Jose Antonio, Ann F. Brown, Darren G. Candow, Philip D. Chilibeck, Stacey J. Ellery, Scott C. Forbes, Bruno Gualano, Andrew R. Jagim, Chad Kerksick, Richard B. Kreider, Sergej M. Ostojic, Eric S. Rawson, Michael D. Roberts, Hamilton Roschel, Abbie E. Smith-Ryan, Jeffrey R. Stout, Mark A. Tarnopolsky, Trisha A. VanDusseldorp, Darryn S. Willoughby, Tim N. Ziegenfuss

**Affiliations:** a Nova Southeastern University, Department of Health and Human Performance, Davie, FL, USA; b University of Idaho, College of Education, Health and Human Sciences, Moscow, ID, USA; c University of Regina, Department of Health and Human Performance, Regina, Canada; d University of Saskatchewan, College of Kinesiology, Saskatoon, Canada; e Monash University, The Ritchie Centre, Hudson Institute of Medical Research and Department of Obstetrics and Gynaecology, Victoria, Australia; f Brandon University, Department of Physical Education Studies, Brandon, Canada; g Universidade de Sao Paulo, Applied Physiology and Nutrition Research Group –School of Physical Education and Sport and Faculdade de Medicina FMUSP, Sao Paulo, Brazil; h Mayo Clinic Health System, Sports Medicine Department, La Crosse, WI, USA; i Lindenwood University, College of Science, Technology, and Health, St. Louis, MO, USA; j Texas A&M University, Department of Kinesiology and Sports Management, College Station, TX, USA; k University of Agder, Department of Nutrition and Public Health, Kristiansand, Norway; l Messiah University, Department of Health, Nutrition, and Exercise Science, Mechanicsburg, PA, USA; m Auburn University, School of Kinesiology, Auburn, AL, USA; n Universidade de Sao Paulo, Center of Lifestyle Medicine, Faculdade de Medicina FMUSP, São Paulo, Brazil; o University of North Carolina, Department of Exercise and Sport Science, Chapel Hill, NC, USA; p University of Central Florida, School of Kinesiology and Rehabilitation Sciences, Orlando, FL, USA; q McMasterChildren’s Hospital, Department of Pediatrics, Hamilton, ON, Canada; r Bonafide Health, Harrison, NY, USA; s Baylor College of Medicine, School of Medicine, Temple TX, USA; t The Center for Applied Health Sciences, Canfield, OH, USA

**Keywords:** Sequel, social media, opinions, research

## Abstract

Creatine monohydrate supplementation (CrM) is a safe and effective intervention for improving certain aspects of sport, exercise performance, and health across the lifespan. Despite its evidence-based pedigree, several questions and misconceptions about CrM remain. To initially address some of these concerns, our group published a narrative review in 2021 discussing the scientific evidence as to whether CrM leads to water retention and fat accumulation, is a steroid, causes hair loss, dehydration or muscle cramping, adversely affects renal and liver function, and if CrM is safe and/or effective for children, adolescents, biological females, and older adults. As a follow-up, the purpose of this paper is to evaluate additional questions and misconceptions about CrM. These include but are not limited to: 1. Can CrM provide muscle benefits without exercise? 2. Does the timing of CrM really matter? 3. Does the addition of other compounds with CrM enhance its effectiveness? 4. Does CrM and caffeine oppose each other? 5. Does CrM increase the rates of muscle protein synthesis or breakdown? 6. Is CrM an anti-inflammatory intervention? 7. Can CrM increase recovery following injury, surgery, and/or immobilization? 8. Does CrM cause cancer? 9. Will CrM increase urine production? 10. Does CrM influence blood pressure? 11. Is CrM safe to consume during pregnancy? 12. Does CrM enhance performance in adolescents? 13. Does CrM adversely affect male fertility? 14. Does the brain require a higher dose of CrM than skeletal muscle? 15. Can CrM attenuate symptoms of sleep deprivation? 16. Will CrM reduce the severity of and/or improve recovery from traumatic brain injury? Similar to our 2021 paper, an international team of creatine research experts was formed to perform a narrative review of the literature regarding CrM to formulate evidence-based responses to the aforementioned misconceptions involving CrM.

## 1. Introduction

Creatine (methylguanidine-acetic acid) was first isolated in the 1830’s [1] but it was not until the 1990’s when creatine monohydrate research really emerged following seminal studies by Harris et al. [2] and Hultman et al. [3] who found that different dosages of CrM influenced plasma and intramuscular creatine levels. To date, there are ≥ 1000 peer-refereed papers involving CrM [4]. Collectively, CrM appears safe [5–7] and is one of the most effective dietary interventions for improving aspects of sport, exercise performance and health across the lifespan [4,8,9]. Despite the wealth of knowledge regarding CrM and robust body of literature supporting the diverse benefits, numerous questions and misconceptions about CrM remain prevalent. To initially address some of these questions and misconceptions, we published a narrative review ‘Common questions and misconceptions about creatine supplementation: what does the scientific evidence really show’ [10]. This paper addressed whether CrM leads to water retention and fat accumulation, is a steroid, causes hair loss, dehydration or muscle cramping, adversely affects renal and liver function, and if CrM is safe and/or effective for children, adolescents, biological females and older adults.

Despite the initial paper, many questions and misconceptions regarding CrM remain prevalent in social media and clinical practice. These include but are not limited to: 1. Can CrM provide muscle benefits without exercise? 2. Does the timing of CrM ingestion really matter? 3. Does the addition of other compounds with CrM enhance its effectiveness? 4. Does CrM and caffeine oppose each other? 5. Does CrM increase the rates of muscle protein synthesis? 6. Is CrM an anti-inflammatory intervention? 7. Can CrM increase recovery following injury, surgery and/or immobilization? 8. Does CrM cause cancer? 9. Will CrM increase urination? 10. Does CrM influence blood pressure? 11. Is CrM safe to consume during pregnancy? 12. Does CrM enhance performance in children and adolescents? 13. Does CrM adversely affect male fertility? 14. Does the brain require a higher dose of CrM than skeletal muscle? 15. Can CrM attenuate symptoms of sleep deprivation? 16. Will CrM reduce the severity of and/or improve recovery from traumatic brain injury?

To address these questions, an internationally renowned team of creatine research experts was again formed to perform a narrative review of the literature regarding CrM to formulate evidence-based responses to misconceptions mentioned earlier involving CrM.

## 2. Can CrM provide muscle benefits without exercise?

Creatine is primarily stored within skeletal muscle and helps facilitate the production of adenosine triphosphate (ATP) within the cell, thereby serving an important role in energy production. As a result, there is some evidence that CrM (without an exercise intervention) can significantly increase total creatine stores and may improve exercise performance [11]. For example, Harris et al. [2] and Hultman et al. [3] showed that a 4.5–10 day CrM protocol at ~ 20 grams/day (referred to as creatine-loading) could increase the total creatine pool (phosphocreatine [PCr] and free creatine; mmol/kg dm) in human muscle. Similar increases in the skeletal muscle creatine pool were seen with the ingestion of 3 grams/day for 28 days [3]. Increased intramuscular creatine levels may help explain some of the improvements observed in the literature on upper- and lower-body strength, anaerobic and physical working capacity, and sprint performance in a hot and humid environment [12–17] in healthy, despite no exercise intervention.

Syrotuik and Bell [18] found that individuals who exhibited a significant increase in total intramuscular creatine (greater than 20 mmol·kg^− 1^ dry weight) after a 5 day CrM loading protocol, termed “responders,” demonstrated significant improvements in strength. However, not all participants exceeded this 20 mmol·kg^− 1^ dry weight threshold. The individual responsiveness to CrM varies depending on several factors, including low resting (pre-supplementation) muscle creatine stores, age, biological sex, type II muscle fiber percentage, physical activity patterns, and habitual dietary intake of creatine [18–20]. Individuals who adhere to vegan, vegetarian, or emphasize plant-based diets tend to have lower baseline creatine levels compared to those on omnivore or carnivore diets due to the absence or reduction in creatine-rich animal-based foods (i.e. seafood, meat) and may respond differently to CrM strategies [21,22]. For example, Watt et al. [23] found that CrM (0.4 grams/kg/day for 5 days or ~ 30 grams/day) significantly increased muscle total creatine content more in vegetarians (*n* = 7; 28 years of age) compared to non-vegetarians (*n* = 7; 23 years of age; consumed ≥ 2 servings of meat/week). Whether this translates to greater improvements in muscle performance without exercise remains to be elucidated. Furthermore, older adults (>65 years) may respond favorably to CrM as there is some evidence that they have reduced skeletal muscle creatine stores compared to younger adults [19] and experience a decline in lean tissue mass, strength, and power over time [24]. Subsequently, CrM may be a viable and effective strategy to offset these age-related consequences of biological aging [19]. Further, CrM alone has resulted in delayed measures of fatigue in older adults. The Physical Working Capacity at Fatigue Threshold (PWCFT), a submaximal cycle ergometer test developed by deVries et al. [25], is useful for evaluating the capacity for physical work, the ability to delay fatigue, and for screening older individuals at risk for sarcopenia [26]. Stout et al. [27] reported that 14 days of CrM (20 grams/day for 7 days followed by 10 grams/day for 7 days) without an exercise intervention significantly increased PWCFT by 15.6% and grip strength by 6.7% in men and women aged 64–86 years. Although this increase in PWCFT was approximately half of the 28% increase observed in the study by deVries et al. [28], which examined 10 weeks of moderate-intensity endurance training, the improvement in PWCFT with CrM alone is still notable, considering the absence of exercise. Forbes et al. [11] performed a narrative review summarizing the small body of research examining the efficacy of CrM without exercise training and concluded that CrM improved measures of fat-free mass, functional ability (i.e. sit-to-stand), and reduced lower-body fatigue. It is important to note that the majority of studies included in this review used a CrM “loading” phase (20 grams/day for 7–10 days) or high daily dosages (17–26 grams/day) of CrM. Studies that did not utilize a CrM loading strategy failed to observe any beneficial effects [29,30].

***In summary, CrM can provide some muscle performance benefits even without exercise. Populations with lower baseline creatine levels, such as vegans and vegetarians, may experience a greater response to CrM.***

## 3. Does the timing of CrM ingestion really matter?

The notion that the timing of CrM (in close proximity to exercise) may influence the physiological adaptations from exercise (primarily resistance training) has historically relied on speculative mechanisms (for reviews, see Candow et al. [31] and Ribeiro et al. [32]). Exercise-induced hyperemia, which is influenced by exercise duration and intensity [33,34], could theoretically affect the delivery and uptake of creatine by skeletal muscle [35,36], though this effect is suppressed shortly (~30 minutes) after exercise cessation. In this context, a single dose of CrM (i.e. 5 grams) may take up to 2 hours to achieve peak levels in the blood following ingestion [2], rendering CrM supplementation immediately before, during, or after a typical resistance training session (e.g. 40–90 minutes) [37] most likely insignificant [32,38]. However, exercise may also modulate sodium (Na^+^)/potassium (K^+^) pump activity, which could contribute to creatine transport and accumulation in active tissues [39,40]. Therefore, similar to hyperemia, pre-exercise CrM-induced elevation in circulating creatine levels could work in conjunction with Na^+^/K^+^ pump activation, thus theoretically favoring tissue uptake and retention. The effect of skeletal muscle contractions on enhanced creatine uptake has been shown over the short term [2]; however, it is unknown whether this may favor longer-term creatine storage past its saturation in skeletal muscle. In theory, CrM timing may be more relevant during the initial stages of a supplementation regimen to enhance creatine uptake and retention until saturation is achieved [38].

Studies directly investigating the timing of CrM are scarce and most lack adequate experimental control (i.e. placebo comparator) and have small sample sizes resulting in low statistical power. Despite Cribb and Hayes [41] showing greater total muscle creatine (and glycogen) levels in bodybuilders supplementing immediately before and immediately after each resistance training session vs. supplementing in the morning and evening; this was a single-blinded experiment using a multi-ingredient supplement containing protein and carbohydrates, limiting further conclusions. More recently, Forbes et al. [42] showed similar effects in muscle strength and regional muscle thickness gains after either pre- or post-resistance training CrM (0.1 grams/kg/day for 8 weeks) in young recreationally active adults (*n* = 10). These results have been further corroborated by Candow et al. [43], Antonio and Ciccone et al. [44], Jurado-Castro et al. [45] and Dinan et al. [46], all showing similar effects in resistance training-induced adaptations, independent of the timing of CrM. In the only study that compared the strategic timing of CrM to a placebo group, Candow et al. [47] reported similar changes in whole-body lean tissue mass (as measured using dual-energy x-ray absorptiometry) and muscle strength (leg press and chest press 1-repetition maximum) after 32 weeks of CrM (0.1 grams/kg or ~ 8 grams immediately before or after each resistance training session; 3 ×/week) in healthy older adults (≥50 years of age). Interestingly, the group who consumed CrM post-exercise had greater improvements in whole-body lean tissue mass over time compared to the placebo group. Additionally, there were no differences in whole-body lean tissue mass increases between the pre-exercise CrM group and placebo group. Certainly, the existing literature on the topic has notable limitations, such as a lack of actual creatine retention measurements, precluding the ability to directly determine the time course of creatine uptake during a resistance training program as a function of its supplementation strategy, thus warranting further research.

***In summary, the current body of evidence does not validate that the timing of CrM is critically important in relation to long-term resistance training, as both pre- and post-exercise CrM seem equally effective in promoting resistance training-mediated gains in lean tissue accretion and muscle performance. Consistent ingestion of CrM during a resistance training program is likely the most important variable to consider.***

## 4. Does the addition of other compounds to CrM enhance its effectiveness?

CrM is effective whether it is co-ingested with other nutrients or not [8,48]. However, there is evidence that creatine uptake into skeletal muscle can be enhanced by glucose ingestion and/or insulin [49–53]. For example, Green et al. [54] reported that the combination of CrM (5 grams), dextrose (18 grams) and glucose (95 grams) promoted greater muscle creatine retention over time. Additionally, Nelson et al. [55] and Roberts et al. [36] showed that CrM had favorable effects on glycogen resynthesis. Burke et al. [56] examined the combined effects of CrM with alpha-lipoic acid (known to enhance glucose uptake) and sucrose compared to CrM and sucrose or CrM alone on intramuscular creatine uptake and retention. Results revealed a significantly greater increase in intramuscular phosphocreatine and total creatine in the CrM, alpha-lipoic acid and sucrose group compared to the other groups. Despite greater increases in creatine uptake and retention over the short term, there is limited evidence that the combination of CrM and carbohydrates or other insulin-sensitizing nutrients lead to greater changes in body composition or performance (i.e. increases in muscular strength), or that it affects the maximal levels attained after a period of supplementation.

In addition to carbohydrates, there has been some interest as to whether protein or a metabolite of the essential amino acid leucine (i.e. beta-hydroxy-beta-methylbutyrate [HMB]) can enhance the effectiveness of CrM. For example, Steenge et al. [49] reported that CrM (4 × 5 grams/day) with either a high amount of carbohydrates (96 grams) or moderate amount of carbohydrates (47 grams) plus 50 grams of protein resulted in greater creatine uptake and retention compared to CrM. Cribb et al. [57] and Cornish et al. [58] reported that CrM combined with carbohydrate, whey protein and/or conjugated linoleic acid (CLA) promoted greater gains in strength and lean tissue mass. There is also some evidence that CrM (0.1 grams/kg/day) and whey protein (0.3 grams/kg/day) increased lean tissue mass (+5.6%) and bench press strength (+25%) after 10 weeks of resistance training in healthy older males compared to CrM or placebo [59]. However, others have not observed the same benefits from CrM and protein [60,61]. In regards to HMB, the change in absolute power achieved at 8 mmol/L of lactate was greater in the CrM plus HMB group compared to either CrM or HMB alone or placebo during a rowing exercise test in endurance-trained young male [62]. In contrast, others have not found greater benefits from the combination of CrM and HMB supplementation on changes in body composition or muscle performance compared to CrM alone [59–61,63].

In regards to other possible potentiating ingredients, CrM with sodium bicarbonate (e.g. 0.3 grams/kg/day) promoted greater performance benefits (e.g. repeated sprint performance, soccer specific performance, and anaerobic performance) than CrM or sodium bicarbonate alone [64–67]. Similarly, CrM with β-alanine provided additive benefits in vertical jump [68], and resulted in greater gains in lean tissue mass in power lifting athletes compared to creatine alone [69]. However, the combination of CrM and β-alanine failed to improve physical working capacity at the neuromuscular fatigue threshold [70], or VO_2_ peak, time to exhaustion, power output or oxygen uptake associated with ventilatory threshold or lactate threshold [71]. Kerksick et al., [72] examined the effects of CrM (20 grams/day for 5 days; 5 grams/day for remaining 23 days) with and without D-pinitol during 4 weeks of resistance training in young resistance-trained males. Intramuscular creatine stores and upper-and lower body muscular strength increased similarly in both groups; however, the CrM group experienced greater improvements in lean tissue and fat-free mass (*p* < 0.05) compared to the CrM and D-pinitol group [72]. Further, Taylor et al. [73] had resistance-trained men (*n* = 47) ingest either 5 grams of CrM + 70 grams of dextrose, 3.5 grams of CrM with 900 mg fenugreek extract or a placebo (70 grams of dextrose) for four days a week for 8 weeks. Participants ingesting CrM experienced similar changes in lean tissue mass and muscle strength compared those on placebo. Similarly, co-ingesting CrM with cinnamon extract (0.5 grams) [74] and Russian tarragon (0.5 grams 30 minutes before ingesting creatine) [75] does not affect whole-body creatine retention, muscle-free creatine, or anaerobic sprint capacity. Additionally, since creatine uptake into the cell increases intracellular hydration, research has evaluated whether co-ingesting CrM with glycerol and water can promote greater fluid retention than glycerol with water [76–78]. These studies generally indicate that co-ingesting creatine (e.g. 11.4 grams/day) with glycerol (2 × 1 grams/kg/day) for 7 days increases total body water [77,79] without altering plasma volume [76] thereby improving thermoregulation during exercise in hot and humid environments [76,77,79].

***In summary, there is some evidence that the combination of CrM with other purported ergogenic compounds (i.e. carbohydrate, protein) can accelerate intramuscular creatine accumulation and potentially increase exercise-related training adaptations.***

## 5. Does CrM and caffeine oppose each other?

Combined ingestion of CrM and caffeine is common due to the daily ingestion of caffeine or combined ingredients in many pre-workout supplements. Due to the popularity and efficacy of both ingredients, as well as their independent mechanistic effects on exercise performance, concurrent ingestion and potential interactions have gained interest. Early research reported that CrM administered in a caffeinated beverage significantly augmented muscle creatine content [80], and the independent pharmacokinetic properties of creatine and caffeine do not appear to be altered when consumed together [81].

Creatine and caffeine have independent ergogenic mechanisms. CrM is known for its ability to increase muscle creatine storage, resulting in greater rephosphorylation of adenosine diphosphate. Whereas caffeine’s positive effects on exercise are attributed to its properties as an adenosine receptor antagonist, potentiation of calcium release from the sarcoplasmic reticulum, along with peripheral effects on substrate utilization and accelerating sodium/potassium pump activity [82]. While these effects are independent, caffeine has been shown to facilitate creatine uptake by stimulating creatine transporters within the sarcolemma [83]. Specifically, caffeine may activate the sodium-potassium ATPase pump, increasing the sodium gradient along the sarcolemma. Creatine transporters rely on extracellular sodium levels, which may be stimulated by the caffeine induced sodium gradient. Thus, some have hypothesized that CrM and caffeine may result in synergistic effects. However, other evidence suggests caffeine may attenuate the ergogenic effects of CrM due to opposing effects on calcium kinetics at the sarcoplasmic reticulum [84], delaying relaxation times and, therefore, reducing exercise performance. Additionally, it has been theorized that co-ingestion of CrM and caffeine would increase symptoms of gastrointestinal distress, which could indirectly reduce performance [83,85]. Vandenberghe et al. [83] examined concomitant ingestion of CrM and caffeine and had three participants report minor gastrointestinal distress (two participants in the CrM only group and one participant in the CrM plus caffeine group). Despite the popularity and efficacy of these two ingredients there is still minimal research exploring the performance effects of co-ingestion with more recent systematic reviews describing 10 relevant studies [86,87]. Available studies have explored acute caffeine intake after CrM loading, or more chronic (>3 days) of combined caffeine and CrM. Collectively, when a single dose of caffeine is consumed acutely (5–6 mg/kg), within 60 minutes prior to exercise, after a CrM loading period (0.3 grams/kg/day for 5–6 days), there seems to be an additive effect on aerobic exercise performance more than CrM alone [87–89]. In contrast, one study to date has demonstrated no additive effect of caffeine (3 mg/kg) after 6 days of CrM loading (0.3 grams/kg/day) [90].

When evaluating chronic caffeine ingestion (300 mg/day or 5 mg/kg/day) along with CrM loading (0.5 grams/kg/day or 20 grams/day) for 3–5 days, no added benefits on maximal force [83], upper or lower body max strength [85,91], or repetitions to fatigue [85] have been reported. One study to date has explored combined ingestion of caffeine (6 mg/kg/day) and CrM (3 grams/day) for 3 days, and demonstrated improved maximal knee extension torque compared to CrM alone [92]. A similar combined dosing strategy involving CrM (20 grams/day for 4 days or 0.5 grams/kg/day for 1 day) and 5 mg/kg/day of caffeine resulted in interference between the two ingredients [83,84], and three other studies reported no effect [81,85,91]. Similarly, combining daily caffeine (300 mg or 5 mg/kg for 3–5 days) during a creatine loading period also does not seem to provide additional exercise benefits [86]. Importantly, despite the lack of effect on performance, there also do not appear to be consistent detrimental effects, thus the ingredients likely do not oppose each other, but also likely do not accelerate ergogenic effects when habitually consumed. The potential opposing effects of caffeine with CrM are more likely influenced by doses of caffeine ≥5 mg/kg, as well as with individuals who have a lower tolerance to caffeine [86].

Additional considerations should be given to the increase in daily creatine use (3–10 grams/day) for benefits beyond just performance. The impact of daily caffeine intake on this daily CrM strategy has not yet been evaluated. Based on their mechanisms, CrM and caffeine likely do not oppose each other when consumed together during the short-term. However, they are also unlikely to provide additional benefits unless CrM has been taken daily long enough to saturate muscle stores, similar to a loading phase.

***In summary, short-term*** **CrM *and caffeine ingestion (<5 mg/kg/day*) *likely does not cause opposing muscle effects. The long-term possible interference effects of CrM and caffeine are unknown. Consider acute caffeine intake after* CrM *loading for potential performance benefits. Chronic caffeine use, combined with* CrM *loading, does not result in greater exercise effects. This combined strategy may increase gastrointestinal distress and may indirectly interfere with performance.***

## 6. Does CrM influence the rates of muscle protein synthesis or breakdown?

The benefits of CrM for increasing muscle mass during resistance training are well established (for reviews see Burke et al. [93], Chilibeck et al. [19], and Forbes and Candow [94]). These skeletal muscle benefits are likely attributable, in part, to creatine’s positive influence on growth-related myogenic transcription factors, satellite cell activity and insulin-like growth-factor I [19]. However, the extent to which CrM influences measures of muscle protein kinetics remain ambiguous. Louis et al. [95] examined the effects of CrM (21 grams/day for 5 days) on the rates of muscle protein synthesis during fasted and fed states in a small group of males (*n* = 6). The authors reported that feeding significantly doubled the rates of muscle protein synthesis by 40%, but CrM did not alter these responses. The same research group also examined the effects of short-term CrM (21 grams/day for 5 days) in conjunction with single-leg resistance exercise [96]. Stable isotope techniques were used to measure myofibrillar and sarcoplasmic protein synthetic rates. The resistance exercise bout increased synthetic rates two- to threefold, but the addition of CrM did not augment these effects. Parise et al. [97] examined the effects of short-term CrM (20 grams/day for 5 days followed by 5 grams/day for another 3–4 days) in healthy young men (*n* = 13) and women (*n* = 14). In men only, CrM reduced the rate of leucine oxidation by 19.6% and decreased the rate of plasma leucine appearance (an indicator of muscle protein catabolism) by 7.5%, indicating a potential anti-catabolic effect. In healthy older men, CrM (0.1 grams/kg/day) during 10 weeks of supervised, whole-body resistance training reduced the urinary excretion of 3-methylhistidine (3-MH; an indicator of whole-body protein catabolism) by 40% compared to a 29% increase for those ingesting placebo [98]. Similarly, healthy older men supplementing with CrM (0.1 grams/kg/day) during 12 weeks of resistance training experienced a significant decrease in 3-MH. However, older women on creatine did not experience the same anti-catabolic effects [99].

***In summary, a small body of research shows that CrM does not increase the rates of muscle protein synthesis. However, there is some existing evidence to support the anti-catabolic effects of CrM in men.***

## 7. Is CrM an anti-inflammatory intervention?

There is preliminary evidence that CrM can attenuate markers of oxidative stress and the inflammatory process in humans, thereby implicating a possible anti-inflammatory role for CrM in a variety of cells and tissue types [100]. However, the mechanisms by which CrM imposes its anti-inflammatory effects are not well understood. The initial occurrence from an acute phase inflammatory response involves the increased secretion of pro-inflammatory mediators such as interleukin-8 (IL-8), tumor necrosis factor-α (TNF-α), and IL-1β [101], and, in response to strenuous exercise, IL-6, interferon-γ (INF-γ), and C-reactive protein (CRP) have been shown to increase [102]. Strenuous exercise has also been shown to increase prostaglandin E_2_ (PGE_2_) instigated by muscle damage [103]. However, CrM (20 grams/day for 5 days) prior to a 3 km race attenuated increases in plasma TNF-α and PGE_2_, compared to placebo, 24 hours following the race [104]. Similarly, CrM for 5 days at a dose of 20 grams/day prior to a half-ironman competition attenuated the increase in plasma levels of TNF-α, INF-γ, IL-1β, and PGE_2_ at 24- and 48-hours following the competition compared to placebo [105]. In an anaerobic exercise scenario, CrM for 7 days at a daily dose of 0.3 grams/kg prior to repeated-sprint exercise resulted in attenuations in TNF-α and CRP at 1-hour post-exercise but had no impact on the oxidative stress markers, superoxide dismutase (SOD) and catalase [106]. Furthermore, Rawson et al. [107] found no effect of 10 days of CrM (0.3 grams/kg/day) on CRP concentrations following acute resistance exercise (5 sets of 15–20 repetitions at 50% of 1-repetition maximum for the back squat exercise) in healthy resistance-trained men. Collectively, these findings suggest that CrM may be anti-inflammatory following aerobic exercise compared to resistance training; however, these results may be related to exercise volume and not exercise modality.

***In summary, short-term*** **CrM *may reduce some markers of inflammation, primarily in response to aerobic-type activities. Further research is needed to determine the long-term mechanistic effects of* CrM *on inflammatory responses to exercise.***

## 8. Can CrM increase recovery following injury, surgery and/or immobilization?

The potential therapeutic utility of CrM for enhancing recovery from injury, surgery and/or immobilization likely relies on its bioenergetic role as an intracellular energy buffer. In these conditions, increased creatine content may offset critical intracellular loss of energy (both in muscle and brain), facilitate the rehabilitation of disuse atrophy and accelerate functional recovery [108,109]. While these assertions seem to be supported by pre-clinical data, clinical trials, which are in their infancy, have shown mixed outcomes. Hausmann et al. [110] found protective effects of CrM in an animal model of spinal cord injury. Twenty adult rats were fed for 4 weeks with or without CrM (5 grams/100 grams of dry food) before undergoing a moderate spinal cord contusion. Rats on CrM showed better posttraumatic locomotor capacity vs. controls 1 and 2 weeks after the injury. Furthermore, histological analysis of the lesion site showed slightly smaller scar tissue in supplemented animals, suggesting a reduced spread of secondary injury. Based on these findings, the authors concluded that CrM could be useful as a neuroprotective aid in cases of elective surgery within the spinal cord. Ozkan et al. [111] investigated the effects of CrM (300 mg/kg) vs. control on the muscle reinnervation of Wistar rats, which had the sciatic nerve experimentally denervated. Rats then had their nerves either repaired with epineural stitches or had the proximal and distal ends of the nerves ligated, with no neural anastomosis. Six months following the procedure, CrM was able to improve the functional properties of denervated muscle (which included histomorphometry and histochemical assessments) in both surgically repaired and unrepaired nerve injuries. These findings led the authors to suggest that CrM could be useful in preventing muscle wasting owing to disuse.

Despite these promising results in animal models, results from human trials are less robust. Hespel et al. [112] conducted a double-blind trial in which young healthy participants (*n* = 22) had their right leg immobilized using a plaster-cast for 2 weeks. Participants then underwent a knee-extension rehabilitation program for 10 weeks, while receiving either CrM (15 grams for the first 3 weeks followed by 5 grams for 7 weeks) or placebo. Prior to and after immobilization, and after 3 and 10 weeks of the rehabilitation stage, quadriceps cross-sectional area, isokinetic knee-extension power and myogenic transcription factors were assessed. The authors observed that CrM promoted muscle hypertrophy during rehabilitative strength training, which was possibly mediated by modulation of the myogenic regulatory factors MRF4 and myogenin expression, both being intracellular proteins that promote muscle growth or through enhanced training capacity due to greater intramuscular PCr and creatine stores. These results expand on the work by Johnston et al. [113] who showed that CrM (20 grams/day for 7 days) preserved lean tissue mass and muscle performance in young healthy adults who volunteered to have their upper limb immobilized (plaster-cast) compared to placebo. Contrary to these findings, Backx et al. [114] failed to demonstrate beneficial effects of CrM in healthy young men (*n* = 30) randomly assigned to receive either CrM (20 grams/day) or placebo for 5 days before one leg was immobilized using a cast for 7 days. Neither the immobilization-induced decrease in quadriceps cross-sectional area nor the decrease in 1-repetition maximum knee extension performance were prevented by CrM, likely suggesting inconsistency in efficacy between short- (1 week) and long-term (3 to 10 weeks) use of this supplementation strategy.

Results are also conflicting in clinical populations. In a crossover fashion, patients with complete cervical-level spinal cord injury (*n* = 16) were given CrM (20 grams/day) or placebo for 7 days. Incremental peak arm ergometry tests were performed following each condition. As compared to placebo, CrM increased VO_2_max, VCO_2_, and tidal volume at peak effort, suggesting potential enhancements in exercise capacity in this population. In contrast, a randomized clinical trial of CrM (20 grams/day for the first 7 days, followed by 5 grams/day for 12 weeks) vs. placebo for patients who underwent anterior cruciate ligament reconstruction (*n* = 60) produced null findings [115]. Quadriceps and hamstring strength and power were measured by an isokinetic dynamometer prior to surgery and at 6 weeks, 12 weeks, or 6 months after surgery. Strength improvements were seen over time as result of the rehabilitation program, but CrM did not enhance the recovery beyond those found from the rehabilitation program alone.

***In summary, mechanistic and pre-clinical data suggest that CrM has the potential to enhance recovery following injury, surgery, or immobilization. However, clinical data remain scarce and conflicting. Confounding factors include CrM protocol (short- or long-term), combination with other therapies (exercise rehabilitation), and type of condition (transitory vs. permanent injury; orthopedic vs. neuromuscular injury). Further, well-powered, randomized controlled trials should address these gaps.***

## 9. Does CrM cause cancer?

A large amount of negative press claiming that CrM causes cancer emerged after a review paper summarized the potential for CrM and creatinine (a metabolic by-product of creatine metabolism) to produce mutagenic/carcinogenic compounds [116]. This paper provided an in-depth review of the literature regarding the potential for CrM and creatinine to be involved in biochemical reactions leading to the production of mutagenic compounds (heterocyclic amines) and concluded that (these compounds, not CrM *per se*), may impose only a minor health risk [116]. In brief, they highlighted the data showing that the consumption of cooked and processed meats (containing creatine and creatinine) led to the production of mutagenic compounds such as amino-imidazo-azaarenes (AIA) and that the amount was proportionate to the amount of creatine/creatinine in meat, the amount of sugar (especially fructose) and amino acids, and the cooking methods. For AIA to become carcinogenic there are a series of other metabolic reactions that must occur before they attain mutagenic (DNA guanidine adducts) potential. Nitrites are formed in the mouth and stomach from dietary nitrates and creatinine can be nitrosylated to form N-methyl-N-nitrosourea, sarcosine and N-nitrososarcosine [117,118]. Some of these nitrosylated compounds can be mutagenic in the Ames test. There is a positive correlation between dietary nitrate/nitrite intake and mortality from gastric cancer mortality, with a lowering of gastric cancer incidence in the United States, co-temporal with a reduction in gastric nitrate/nitrite load from 1925 to 1981 [119,120]. Collectively, this body of literature supports the recommendation that people should limit the consumption of highly processed, overcooked (very prolonged cooking, blackened [i.e. barbeque; BBQ] flesh containing creatine and creatinine [meat, fish]). Interestingly, when pigs were fed high doses of CrM (50 grams/day), there was no increase in the levels of heterocyclic amines in the meat from supplemented vs non-supplemented animals [121]. Importantly, there is no evidence that CrM at typical recommended levels (i.e. 3–5 grams/day) would lead to the production of such carcinogenic compounds without simultaneously being exposed to high temperatures, amino acids, nitrites and sugars. Indeed, CrM at 7 grams/day for 7 days followed by ≤ 5 grams/day for 23 days does not lead to an increase in carcinogenic heterocyclic amines in young males and females [122]; an epidemiological study in > 7000 men and women found that a lower dietary creatine intake was associated with a slightly higher risk of cancer (which remained after controlling for moderate physical activity and other lifestyle factors) [123]; and most pre-clinical studies showing *in vivo* carcinogenesis used heterocyclic amines at much higher amounts than those found in cooked/processed meats.

A second “peak” in the speculation that CrM causes cancer resulted from a paper looking at cancer metastasis in a murine (rodent) model and concluded that CrM in cancer survivors promotes cancer metastasis [124]. In brief, this study found that cancer cells upregulate glycine amidino transferase (*GATM*) expression to increase intra-cellular creatine production and that blocking this up-regulation attenuated metastasis. They also found that CrM (5 % wet-weight; w/w) did not alter primary colonic tumor growth (and even mildly suppressed it) but enhanced the metastases to the liver [124]. There are several caveats to this study including that the 5 % w/w CrM supplement dose used is equivalent to ~ 28 grams of CrM/day in humans or 5–9 times the typical recommended CrM long-term dose of 3–5 grams/day. Secondly, the metastases were in the liver and liver inflammation appears to be a species specific side effect of CrM in mice but not in rats or humans [125]. Furthermore, it is clear that cancer cells are voracious consumers of energy and promiscuous in terms of the type of energy source needed to support rapid growth and metastasis. The Warburg effect is a cancer cell specific upregulation of glycolytic flux that increases the flux of carbon from glucose to 3-phosphoglycerate toward DNA synthesis through the serine-glycine-one carbon (SGOC) pathway [126]. Due to the high energetic demand from rapidly growing tumor cells, it is also not surprising that many tumor cells express high level of cytosolic creatine kinase (CK-BB) [127], and ubiquitous mitochondrial CK (CKMT1) [128,129]. In fact, this phenomenon was targeted using cyclocreatine to block the reaction and attenuate energy transduction and hence carcinogenesis in several studies [130,131]. Another complicating issue is that tumor cells can alter aspects of creatine metabolism when they transition from primary tumors to metastatic tumors with the former expressing high levels of CKMT1 and the latter showing down-regulation [129]. The down-regulation of CKMT1 is associated with higher levels of reactive oxygen species that promote metastasis dependent expression of adhesion and matrix degradation proteins that are protected using anti-oxidants [129]. As a consequence of the high metabolic demand of cancer cells, the changing dynamics of metabolism between and within different types of cancer cells as they transition from a primary to a metastatic cancer makes conclusions about when a metabolic substrate could be deleterious, neutral or beneficial to a patient with a primary or metastatic tumor very complicated. Indeed, other studies have shown that creatine does not promote tumor growth or proliferation [132], or is protective in some cancer models [133–135], possibly by up-regulating the energy supply to anti-cancer T-cells [133,136].

A final consideration regarding CrM and cancer comes from the strong evidence that CrM can protect against several deleterious health consequences from tumor (i.e. cancer cachexia) and/or the treatment (i.e. chemotherapy). Cancer cachexia refers to the loss of lean tissue mass in response to the flux of energy to the growing tumor, lower energy intake due to the tumor and/or chemotherapy and in response to cytokines that alter metabolism and appetite. Given the well established benefits of CrM on measures of lean tissue mass and performance [137–140], it is not surprising that several studies are planned to evaluate the benefits of exercise training and CrM in cancer survivors [141–143]. Many studies have shown the benefits of CrM with or without exercise training to attenuate the deleterious effects of chemotherapy (doxorubicin) upon lean tissue mass and function [144–148], and one study found that children treated with prednisone for acute lymphoblastic leukemia had lower body fat when supplemented with CrM [149].

***In summary, evidence-based research does not support that CrM (3–5 grams/day) in humans increases the formation of carcinogenic compounds or cancer risk (primary or metastasis). It is likely to be beneficial to help protect and/or recover from the skeletal muscle and body composition issues associated with cancer per se and/or the effects of chemotherapy. It is prudent to limit the intake of highly processed and/or overcooked meats/fish (i.e. BBQ) to lower the risk of gastric cancers.***

## 10. Will CrM increase urine production?

Urine production is a finely regulated process primarily controlled by the kidneys through intricate mechanisms involving filtration, reabsorption, and secretion [150]. Key factors influencing urine production include the glomerular filtration rate (GFR), hormonal regulation, fluid intake, solute load, and renal perfusion pressure. These factors collectively determine the volume and concentration of urine produced by the body to maintain fluid and electrolyte balance [150]. One common misconception is that CrM directly leads to increased urine production. However, scientific evidence (though very few studies use urine volume as a primary outcome variable) suggests that it is not CrM that causes a rise in urine production but rather the associated increase in water or fluid intake often consumed at the same time with CrM, typically in conjunction with exercise training. Kreider et al. [151] examined 98 Division IA college football players’ urine output during a 21-month period of CrM. Compared to placebo, CrM resulted in similar urine outcome variables over time. Increased fluid intake, often encouraged alongside CrM, is likely the main reason urine production increased. When more fluids are consumed, the kidneys upregulate GFR activity to maintain the body’s fluid and electrolyte balance by filtering the excess water out of the bloodstream and into the urine [150]. This process is regulated by antidiuretic hormone (ADH), which controls the amount of water reabsorbed by the kidneys. Higher fluid intake reduces ADH levels, leading to less water reabsorption and consequently, increased urine production. This physiological response tightly regulates fluid homeostasis, resulting in more frequent urination. It is important to note that creatine is efficiently metabolized by the body, with daily intake and excretion being approximately equal [152]. Therefore, the idea that CrM itself leads to a significant increase in urine production is unfounded and not supported by scientific evidence.

***In summary, while CrM may result in higher fluid intake, leading to increased urine production, CrM itself does not independently drive changes in urine volume.***

## 11. Does CrM influence blood pressure?

There has been speculation that CrM might increase blood pressure due to a number of factors, including fluid retention within cells and increased stress on kidney function. In addition, creatine kinase, the enzyme involved in the process by which PCr re-phosphorylates ADP to ATP is high in resistance arteries and may be involved with vasoconstriction to increase blood pressure [153]. Serum creatine kinase, as a surrogate for tissue creatine kinase, is associated with increased blood pressure [153]; however, the concentration of this enzyme in blood is not a direct indication of creatine concentration in tissue, but rather an indication of plasma membrane damage (majority coming from skeletal muscle). Despite these concerns, relatively high-dose (i.e. 10–20 grams/day) but short term (i.e. 5–31 days) CrM in young, healthy males and females did not affect blood pressure [154–156]. Most importantly, a systematic review of clinical populations (i.e. patients with heart failure, ischemic heart disease, or myocardial infarction) found no impact of CrM (again at relatively high doses, i.e. 20 grams/day for up to 6 weeks) on blood pressure [157]. In a recent two-year intervention (which focused on changes in bone), postmenopausal women (mean age ~59 years) were randomized to receive a relatively high dose of CrM (0.14 grams/kg/day) or placebo [158] and participants at one of the research sites had blood pressure measured before and after the intervention. For those who completed the intervention (*n* = 60 on CrM, *n* = 52 on placebo) there was no difference between groups for changes in either systolic (CrM = 121 ± 15 to 124 ± 11 mmHg; placebo = 116 ± 13 to 121 ± 15 mmHg; *p* = 0.34) or diastolic (CrM = 77 ± 8 to 76 ± 7 mmHg; placebo = 75 ± 8 to 76 ± 9 mmHg; *p* = 0.65) blood pressure. Studies of CrM in people who were hypertensive at baseline are missing from the literature; however, one study in spontaneously hypertensive rats found no effect of CrM (5 grams/kg/day for 9 weeks) on blood pressure [159]. Finally, there is some research indicating CrM might be effective for reducing the blood pressure response to a resistance-training session: Three weeks of CrM (10 grams/day) in young males reduced the acute blood pressure response to resistance-training compared to placebo [160]. It was speculated that an improvement in anaerobic metabolism with CrM would attenuate production of metabolic by-products such as lactate or ammonia, which might stimulate muscle metaboreceptors involved in activation of the sympathetic nervous system [160].

***In summary, there is no evidence that short-term or chronic CrM adversely affects blood pressure.***

## 12. Is CrM safe to consume during pregnancy?

Regulation of creatine metabolism may be important in all aspects of reproduction, from fertilization to pregnancy and fetal growth [161–167], labor and birth [168], breastfeeding [169], and early childhood development [170]. This increasing body of research is raising questions about optimal dietary creatine intake during pregnancy and whether CrM may benefit certain populations [171,172], including infants born preterm [173]. In particular, studies are ongoing to explore the potential of CrM during pregnancy to increase fetal creatine reserves and support energy homeostasis during periods of mild to severe hypoxia-ischemia, potentially preventing major complications like perinatal hypoxic-ischemic encephalopathy (HIE). Initial findings from pre-clinical models have been promising [174]. An interesting aspect of CrM for improving newborn outcomes is its use as a prophylactic agent in high-risk pregnancies [175]. This approach addresses the challenge of identifying pregnancies at risk of acute hypoxic events [176]. However, it does raise questions about potentially exposing healthy fetuses to high creatine concentrations throughout the antenatal period [177].

Generating data on the safety of CrM during pregnancy, in addition to establishing its efficacy, has been integrated into the design of recent pre-clinical animal studies. In the spiny mouse model of intrapartum hypoxia, where pregnant dams were fed a 5% w/w CrM from mid-gestation until delivery (a period of 18 days) [178], offspring exposed to increased levels of creatine *in uter*o were followed through to adulthood with no reported impact on body growth, renal, skeletal muscle or diaphragm structure or function [179–181]. In addition, a study conducted in non-pregnant and pregnant spiny mice focused on the impact of CrM on maternal creatine homeostasis, body composition, capacity for *de novo* creatine synthesis and renal excretory function found no sustained negative impacts on maternal physiology [182]. Comprehensive data on fetal well-being has also been reported from chronically instrumented fetal sheep supplemented with a continuous intravenous infusion of 6 mg·kg^−1^·h^−1^ of CrM for 13 days at the cerebral development equivalent gestational age of 34 to 36 weeks of human pregnancy. The 5-fold increased circulating creatine concentrations achieved with direct fetal supplementation had no impact on cardiovascular or systemic arterial blood gas parameters throughout the supplementation period and no change in fetal body or organ weights at postmortem [183]. This CrM regime also did not negatively impact cerebral interstitial concentrations of lactate, glutamate or hydroxy free radicals in the near-term fetal brain [184]. At a cellular level, *in utero* creatine exposure was linked to transcriptional changes in genes associated with anti-apoptotic pathways and mitogenesis, particularly in the fetal hippocampus, but this was not directly linked to any changes in cell death, mitochondrial respiration or mitochondrial complex density [185]. Changes in genes associated with innate immunity in the cortical gray matter and striatum have also been noted, including an upregulation of major inflammatory mediators including IL-6, IL-1β, TNFα, and prostaglandin synthase. Whether these changes indicate CrM produces a pro-inflammatory environment is yet to be ascertained. However, the same study did not observe increases in the presence of microglia and astrocytes nor increased markers of oxidative stress at a protein level [185,186]. Of note, increased myelin basic protein immuno-positive area coverage in the subcortical white matter observed in fetal sheep indicate a potential for *in utero* CrM to enhance or speed up myelination in the developing brain. A similar finding has been reported in male rat pups born to dams supplemented with 1% CrM in their drinking water for the final 10 days of pregnancy. Electrophysiological recordings from hippocampal pyramidal neurons of these rat pups at postnatal day 14–21 displayed increased excitability and enhanced long-term potentiation, despite increased creatine exposure being ceased at birth [187]. In follow-up assessments, these neurological changes observed in young rats persisted, suggesting improved synaptic plasticity into adulthood [188]. While the subtle changes observed in the brain following *in utero* creatine exposure to date do not suggest a negative impact on offspring welfare or cognitive abilities, it remains important to thoroughly characterize any shift in neurodevelopment brought about by CrM during pregnancy with continued pre-clinical evaluation.

The translation of CrM as a pregnancy intervention is still in its infancy, with initial pharmacokinetic and tolerability studies underway (ACTRN:12620001373965). As such, there is currently no direct evidence available from well-designed and executed randomized controlled clinical trials (RCTs) on the safety and tolerability of CrM during human pregnancy. However, a recent systematic review and meta-analysis of CrM in female-only populations of reproductive age assessed the safety outcomes [189]. This study reviewed the data from 29 studies that collectively consented 951 participants ranging from 16 to 67 years of age that received 1–30 grams of CrM per day for between 4 days to 365 days. No deaths or serious adverse events (defined as any outcome that causes life-threatening events; requirement for hospitalization or prolongation of existing hospitalization; persistent or significant disability; or any events considered medically important) were reported in any of the trials reviewed. When stratified by dosing regimens, meta-analyses confirmed that there were also no significant differences in minor adverse events between CrM and control groups, including gastrointestinal events, blood and urine biomarkers of renal and hepatic function, or weight gain.

***In summary, preliminary research involving animal models suggest that*** CrM ***during pregnancy does not negatively affect the mother or offspring. However, there is currently no direct evidence available from well-designed and executed randomized controlled clinical trials on the safety and tolerability of*** CrM ***during human pregnancy.***

## 13. Does CrM enhance performance in adolescents?

In conjunction with the substantial amount of evidence that supports the performance-enhancing abilities of CrM for various adult populations [8,190,191], the body of scientific literature regarding the benefits of CrM for children and adolescents continues to grow (Table 1). To date, many of the studies involving adolescents have focused on swimming and soccer performance [202,203]. Most studies employed a short-term (4–7 days) CrM loading dose of 20–25 grams/day, followed by a CrM maintenance dose of 5 grams/day. Grindstaff et al. [193] were one of the first to evaluate the effects of CrM on sport-specific performance in adolescent male swimmers and reported significant improvements in sprint swimming performance after nine days of CrM at a daily dose of 21 grams. In a similar study, Juhasz et al. [194] reported significant improvements in power output and 100 m sprint swimming performance following CrM (20 grams for 5 days) in elite junior male swimmers.

**Table 1.** Summary of the effects of creatine supplementation in adolescents.

Author Year (Country)	Subjects	Study Design	Duration	Dosing Protocol	Primary Variables	Results	Adverse Events
*Swimming*							
Dawson et al. [192] (Australia)	10 males, 10 females (16.4 ± 1.8 years)	Matched, placebo-controlled	4 weeks	20 g/day (5 days) 5 g/day (22 days)	Sprint swim performance and swim bench test	↑ swim bench test performance	None reported
Grindstaff et al. [193] (USA)	18 (11 F, 7 M) adolescent swimmers (15.3 ± 0.6 years)	Randomized, double-blind, placebo controlled	9 days	21 g/day	Sprint swim performance; arm ergometer performance	↑ sprint swimming performance	None reported
Juhasz et al. [194] (Hungary)	16 male fin swimmers (15.9 ± 1.6 years)	Randomized, placebo-controlled, single-blind trail	5 days	20 g/day	Average power, dynamic strength (swim based tests)	↑ anaerobic performance ↑ dynamic strength	None reported
Theodorou et al. [195] (United Kingdom)	10 elite female (17.7 ± 2.0 years) and 12 elite male (17.7 ± 2.3 years) swimmers	Randomized, double-blind, placebo-controlled	11 weeks	25 g/day (4 days) 5 g/day (2 months)	Swimming interval performance	↑ interval performance following loading phase ↔ long-term improvements after maintenance dose	None reported
Theodorou et al. [196] (United Kingdom)	10 swimmers (6 M, 4F) (17.8 ± 1.8 years)	Randomized, double-blind trial	4 days	20 g/day of CrM or 20 g/day of CrM +100 g of carbohydrates per serving	High-intensity swim performance during repeated intervals	↑ mean swim velocity for all swimmers ↔ swim velocity in Cr + Carbohydrate condition	Gastrointestinal discomfort in Cr + Carbohydrate group only
*Soccer*							
Claudino et al. [197] (Brazil)	14 male elite soccer players (18.3 ± 0.9 years, 69.9 ± 8.8 kg)	Randomized, double-blind, placebo-controlled	7 weeks	20 g/day (1 week) 5 g/day (6 weeks)	Lower limb muscle power via countermovement vertical jump	↔ lower body power	None reported
Mohebbi et al. [198] (Iran)	17 soccer players (17.2 ± 1.4 years, 61.7 ± 1.4 kg)	Randomized, double-blind, placebo-controlled	7 days	20 g/day	Repeated sprint test, soccer dribbling performance and shooting accuracy	↑ repeat sprint performance ↑ dribbling performance	None reported
Ostojic et al. [199] (Yugoslavia)	20 male soccer players (16.6 ± 1.9 years, 63.6 ± 5.6 kg)	Matched, placebo-controlled	7 days	30 g/day	Soccer specific skills tests	↑ dribble test and endurance times ↑ sprint power test and countermovement jump	None reported
Yanez-Silva et al. [200] (Brazil)	19 male soccer players (17.0 ± 0.5 years, 66.8 ± 3.2 kg)	Matched, double-blind, placebo-controlled	7 days	0.03 g/kg/day	Muscle power output (Wingate anaerobic power test)	↑ peak and mean power output ↑ total work	None reported
Ojeda et al. [201] (Chile)	28 male soccer players (17.1 ± 0.9 years, 68.5 ± 6.0 kg)	Randomized, double-blind, placebo-controlled	14 days	0.3 g/kg/day	Muscle power assessed after fatigue induction	↑ in bar velocity and power in creatine group led to significant differences between groups after supplementation.	No

↔ = Creatine supplementation resulted in no significant (*p* < 0.05) change; ↑ = Creatine supplementation resulted in a significant increase (*p* < 0.05) over control. CrM = creatine monohydrate; g/day = grams per day. Adapted from Jagim et al. [202].

For soccer-specific performance, Mohebbi et al. [198] reported improvements in repeat sprint performance and dribbling performance after seven days of CrM supplementation (20 grams/day)) in adolescent male soccer players. Similarly, Ostojic et al. [199] reported improvements in dribble test and endurance times along with improvements in sprint power and countermovement jump performance following seven days of CrM (30 grams/day) in adolescent male soccer players.

Research has also explored alternative applications of CrM beyond direct measures of sports performance. For example, Ojeda et al. [201] recently reported that CrM (0.3 grams/kg/day for 14 days) led to an improved ability to maintain muscle power following the induction of intrasession fatigue in young (17 years of age) male soccer players. Additionally, Juhasz et al. [204] observed greater improvements in segmental lean mass and plantar flexion torque following limb immobilization in adolescent swimmers recovering from tendon overuse injuries after CrM (20 grams/day for 5 days followed by 5 grams/day for 37 days).

***In summary, CrM can improve measures of sports-specific activities as well as similar indices of physical performance such as power or sprint speed in adolescents. Research in female adolescent athlete populations is significantly lacking. Long-term RCT’s designed to examine changes in training adaptations in adolescent populations from*** ***CrM*** ***are needed.***

## 14. Does CrM adversely affect male fertility?

The notion of CrM having detrimental effects on male fertility seems to be propagated by dubious sources. This speculation often stems from studies involving multi-component bodybuilding supplements that contain various ingredients, including anabolic steroids, which can potentially impair sperm function [205,206]. Furthermore, anecdotal claims in public media suggest that CrM may induce dehydration, leading to impaired sperm production and function, or elevate testosterone levels, which could negatively impact male fertility. However, contrary to these claims, CrM actually improves water retention [207], and the majority of studies indicate that it has no effect on testosterone levels (for an in-depth review, see Antonio et al., [10]). Hence, the anecdotal claims regarding the harmful effects of CrM on male fertility lack validity.

Creatine may play a significant role in sperm viability. Semen, being a high-energy-demanding fluid, exhibits relatively high creatine content in both spermatozoa and seminal plasma (up to 15 mm) [208], comparable to levels found in other energy-demanding cells. The testes express a unique tissue-specific membrane transport protein for creatine (CT2) [209], underscoring the importance of creatine utilization for male reproductive bioenergetics. Several preclinical and clinical studies have demonstrated that low semen creatine levels are associated with reduced sperm quality (for a detailed review, see Ostojic et al., [210]). This suggests that restoring normal creatine homeostasis in spermatozoa, possibly through CrM, could be a potential target for improving sperm quality.

Preliminary studies indicate that creatine may enhance human sperm viability. Incubating semen or migrated sperm fractions with creatine phosphate has shown to significantly improve sperm motility and velocity in normospermic donors [161]. These effects occur rapidly, with full improvements in sperm velocity and motility achieved within one minute. The authors suggest that adding creatine phosphate to insemination media could enhance the fertilizing capacity of sperm during *in vitro* fertilization or gamete intrafallopian transfer procedures. Animal studies support these findings, demonstrating the beneficial effects of creatine and creatine analogs on sperm capacitation in mice [211], fertilization ability in boars [212], and semen quality and fertility in broiler breeder roosters [213]. However, no effects of creatine on *in vitro* capacitation-related events were found in frozen equine sperm [214]. An intriguing cross-sectional study suggests that semen concentration and total sperm count may tend to be higher in healthy men who currently consume protein supplements (with 44% reporting CrM use or creatine-protein combinations) compared to former users and never users [215]. Currently, there are no human studies available investigating the effects of CrM on indices of male fertility in normospermic and/or oligospermic men.

***In summary, existing evidence does not suggest that*** CrM ***negatively impacts male fertility. In fact, preliminary findings indicate that exposure to creatine may improve human sperm motility and velocity in normospermic men under*** **in vitro *conditions.***

## 15. Does the brain require a higher dose of CrM than skeletal muscle?

The optimal dose and requirements for CrM may differ between skeletal muscle and brain tissue [216,217]. There is a well-established body of literature examining the effects of various dosages of CrM on skeletal muscle uptake and retention (for review see [140,216]). Pioneering work in the early 1990’s by Drs. Roger Harris and Eric Hultman demonstrated that ingesting 20 grams/day of CrM separated into four equal doses was able to elevate intramuscular creatine stores ~ 18% within 6 days, which was maintained with 2 grams/day [3,218]. Furthermore, a much lower dose of CrM (3 grams/day) was able to elevate creatine stores to a similar degree after 28 days of supplementation compared to the CrM loading phase (20 grams/day for 6 days). Importantly, CrM elevates plasma creatine (5 gram dose = >500 mmol/L increase), which can then be taken up into energetically demanding tissues (e.g. muscle and brain) against a concentration gradient via a sodium-chloride creatine-specific transporter protein (SLC6A8) [40,116]. Creatine transport into cells is both sodium and insulin-dependent [219]. Several studies from Dr. Paul Greenhaff’s laboratory have shown that insulin augments creatine uptake into muscle and that CrM co-ingestion with either carbohydrates or protein (which both are insulinogenic) results in greater intramuscular creatine retention in the short-term [49,54,219].

In contrast to skeletal muscle, there is less evidence and understanding regarding the optimal CrM dosing protocol to increase brain creatine levels [216]. A few studies have shown that high-dose CrM (acute ingestion of 0.35 grams/kg; ≥20 grams/day or 0.3 grams/kg/day for at least 7 days) or lower-dose CrM (4–5 grams/day for several months) can increase total brain creatine levels in young and older adults [220–224], while others have found no effect after 7 days with ~ 20 grams/day [225]. Importantly, CrM increases brain creatine levels by ~ 5–10% compared to a ~ 20–40% increase in skeletal muscle [217,226]. These divergent responses may be associated with the endogenous synthesis of creatine in the brain, lack of creatine transport kinetics at the blood-brain barrier, and dosage and duration of CrM [217]. Skeletal muscle relies solely on exogenous creatine (i.e. dietary creatine and creatine synthesized in the liver), while the brain has the ability for *de novo* synthesis. The synthetic pathway involves three amino acids: arginine, glycine, and methionine and two enzymes: L-arginine: glycine amidinotransferase (AGAT) and guanidinoacetate methyltransferase (GAMT). The brain appears to rely on its own creatine synthesis under normal resting conditions. For example, CrM does not alter brain energetics or cognition in young, healthy adults, even with higher doses (e.g. 20 grams/day for 6 weeks) [227]. Further, the capacity to uptake creatine from the blood appears to be limited due to a small number, or lack thereof, of SLC6A8 transporters at the blood-brain barrier [228]. There is speculation that the brain does not necessarily rely on circulating creatine to maintain homeostasis, therefore higher doses of CrM over longer periods might be required to elevate brain creatine levels. However, this concept remains to be elucidated as previous creatine trials fail to demonstrate a dose and/or duration response relationship. For example, adolescent females on medication for major depressive disorder supplemented with either 2, 4, or 10 grams/day of CrM or placebo for 8 weeks [229]. Mean frontal lobe phosphocreatine increased by 4.6, 4.1, and 9.1% in the 2, 4, and 10 grams/day of CrM groups, respectively. Since there were no differences in the 2 and 4 grams/day of CrM groups, there does not appear to be any clear linear response, however the higher dose CrM group (10 grams/day) experienced twice the increase in brain creatine stores. Furthermore, Dechent et al. [221] had participants ingest 20 grams/day of CrM for 4 weeks and monitored brain creatine levels on a weekly basis. Results showed that total brain creatine levels increased early in the CrM period, decreased in week 3, and then increased again in week 4. Solis et al. [225] found that CrM (0.3 grams/kg/day for 7 days) did not alter brain creatine levels in young or older adults. Overall, these findings are challenging to interpret, but highlight that there is no clear dose-time relationship regarding CrM and brain creatine levels.

***In summary, it is unclear whether the brain requires higher doses of*** **CrM *compared to skeletal muscle. It is well-established that a wide range of* CrM *protocols (20 grams/day with and without a maintenance dose, 3–5 grams/day) can increase skeletal muscle creatine stores. There is some evidence that different* CrM *dosing protocols (acute ingestion of 0.35 grams/kg; ≥20 grams/day or 0.3 grams/kg/day for at least 7 days) or lower-dose CrM (4–5 grams/day for several months) can increase brain creatine levels. However, a CrM dose and time-response relationship (if any) remains to be determined.***

## 16. Can CrM attenuate symptoms of sleep deprivation?

Lack of sleep adversely affects cognitive function, motor skills, and mood, partly because of reduced creatine levels in the brain. Hence, it has been posited that CrM could alleviate these detrimental effects of sleep deprivation [220,226]. Nevertheless, there is a scarcity of data on the effects of CrM on sleep. McMorris et al. [230] assessed the effects of CrM, sleep deprivation, and mild exercise on cognitive and psychomotor performance, mood, and plasma concentrations of catecholamines and cortisol. In this double-blind, placebo-controlled trial, research participants (21 years of age) consumed 5 grams of CrM or placebo four times daily for one week. Participants underwent various tests including random movement generation (RMG), verbal and spatial recall, choice reaction time, static balance, and mood assessment at baseline (0 h) and after 6, 12, and 24 hours of sleep deprivation, interspersed with intermittent exercise. Blood samples were collected at 0 and 24 hours to measure plasma concentrations of catecholamines and cortisol. Results revealed that after 24 hours, the CrM group exhibited significantly less deterioration in RMG, choice reaction time, balance, and mood compared to baseline. However, there were no significant differences between groups in terms of plasma catecholamines and cortisol concentrations. In a subsequent study by McMorris et al. [231], they discovered that in the context of moderate-intensity exercise during sleep deprivation, CrM (i.e. 20 grams/day for 7 days) influenced the performance of intricate central executive tasks during 36 hours of sleep deprivation in young males (21 years of age). Furthermore, Cook et al. [232] explored how sleep deprivation, with or without the immediate intake of caffeine or CrM, affected performance during a repetitive rugby passing skill [232]. Ten top-tier rugby athletes (21 years of age) underwent 10 trials of a basic rugby passing skill test (i.e. 20 repetitions per trial) after becoming accustomed to the task. During 5 trials, participants slept between 7–9 hours, while during the other 5 trials, they experienced sleep deprivation, sleeping only 3–5 hours. Prior to each trial, participants received either: placebo pills, 50 or 100 mg/kg of CrM, or 1 or 5 mg/kg of caffeine. Saliva samples were collected before each trial and analyzed for levels of salivary free cortisol and testosterone. The CrM dose would be equivalent to 3.75–7.50 grams of CrM for a 75-kilogram individual. Sleep deprivation under the placebo condition led to a substantial decline in skill performance accuracy on both the dominant and non-dominant passing sides. However, there was no decline in skill performance observed with caffeine doses of 1 or 5 mg/kg, and there was no significant difference in the effects between these two doses. Similarly, no impairment was observed with CrM at doses of 50 or 100 mg/kg, and there was no significant difference in the performance effects between these two doses. Salivary testosterone levels were unaffected by sleep deprivation. Thus, CrM appears to ameliorate the effects of sleep deprivation [232]. In an intriguing investigation by Gordji-Nejad et al. [222], participants (23 years of age) were orally administered a high single dose of CrM (0.35 g/kg) while performing cognitive tests during 21 hours of sleep deprivation. Results showed that a high single dose of CrM can partially reverse metabolic alterations and fatigue-related cognitive deterioration [222]. In contrast, Rawson et al. [233] found no beneficial effects from CrM (0.03 grams/kg/day) on measures of cognitive processing in young adults (21 years of age) who were not sleep-deprived [233].

***In summary, preliminary evidence suggests that*** CrM ***may have a positive effect on cognitive processing under conditions of sleep deprivation in young adults. However, there is no evidence that creatine supplementation improves cognition under conditions of adequate sleep.***

## 17. Will CrM reduce the severity of or improve recovery from traumatic brain injury?

It is becoming more widely known that increasing brain creatine through CrM improves aspects of brain health, such as cognitive processing, under both resting and especially under stressed (e.g. disease, sleep deprivation, etc.) conditions (reviewed in [217,220,234]). Traumatic brain injury (TBI) represents a unique challenge to brain health, but there is some evidence that CrM may be of benefit. Following TBI, there is increased energy need coupled with decreased energy availability, including reduced brain creatine [235,236], which creates a cellular energy crisis. In addition to the potential of CrM to help mitigate the energy drain created by brain injury, CrM may positively impact other features of TBI including: membrane disruption leading to calcium influx, nerve damage, mitochondrial dysfunction, oxidative stress, and inflammation (reviewed in [237]).

Animal models have been used to examine the ability of CrM to serve as a prophylactic nutrient to moderate the damage of TBI. Sullivan et al. [238] reported that 3 or 5 days of prophylactic creatine ingestion (3 mg/gram/day) reduced cortical damage 21% and 36%, respectively, in mice following an experimentally induced TBI. Similarly, rats fed a creatine-enriched diet prior to experimentally induced TBI demonstrated a 50% reduction in cortical damage. It is, however, difficult to study the preventative effects of CrM on TBI in humans, as concussing research volunteers is unethical and identifying concussed volunteers cannot happen until after the injury. The effects of post-brain injury CrM in children have been studied by Sakellaris et al. [239–241]. Reportedly, 6 months of CrM (0.4 grams/kg/day) in children and adolescents (*n* = 39: 1–18 years of age) with a TBI revealed many benefits, including decreased duration of post-traumatic amnesia, intubation time, and intensive care unit stay; and improved disability, recovery, self-care, communication, locomotion, sociability, personality and behavior, and neuro-physical and cognitive function [239,240]. CrM improved post-traumatic headaches, dizziness and fatigue, commonly reported as lingering problems of TBI. Dysregulation in brain energy metabolism following TBI needs further study, as TBI-related changes in brain metabolites are influenced by brain region and time [242]. Compelling data from Alosco and colleagues [243] showed that persistent, longer-term changes to cognitive, behavioral, and mood symptoms are related to reduced brain creatine in retired players from the National Football League (aged 40 to 69 years) who had experienced repetitive head impacts years earlier during their career. Furthermore, several RCT’s designed to investigate the potential benefits of CrM on recovery from TBI are currently underway (see [244], Clinicaltrials.gov ID NCT06208813, NCT05562232, NCT0558906). Additionally, a recent review by Ostojic et al. (20024) found that increased dietary creatine intake was associated with reduced circulating levels of neurofilament light chain levels, which is a common marker of neuronal damage in humans. Therefore, dietary creatine intake may exert protective effects of future neuronal injury [245].

***In summary, the small body of research to date involving animal and patient populations suggest that*** CrM ***can potentially reduce severity of and/or improve recovery from TBI. In lieu of data from large RCT’s, for best practice, the totality of evidence suggests that*** CrM ***for individuals at high-risk of TBI, such as athletes and military personnel, is sensible.***

## 18. Conclusions

Based on our scientific evaluation of the literature, we conclude that:

1. CrM may provide benefits to skeletal muscle without exercise. Populations with lower baseline creatine levels, such as vegans and vegetarians, may experience a greater response to CrM.
2. The timing of CrM does not appear to be a limiting factor on the ergogenic effects of exercise training adaptations. Consistent CrM during an exercise training program is likely the most important variable.
3. The co-ingestion of CrM with other compounds (i.e. carbohydrates, protein) may accelerate the increase in muscle creatine levels and improve exercise performance.
4. Short-term creatine and caffeine ingestion (<5 mg/kg/day) likely do not cause opposing effects. Consider acute caffeine intake after CrM loading for potential performance benefits. Chronic caffeine use, combined with CrM does not result in greater exercise effects. This combined strategy may increase gastrointestinal distress and may indirectly interfere with performance.
5. CrM does not increase the rates of muscle protein synthesis. However, there is some existing evidence to support the anti-catabolic effects of CrM in men).
6. CrM changes some inflammatory markers following long-duration aerobic type exercise.
7. CrM has the potential to enhance the recovery following injury, surgery or immobilization.
8. Evidence-based research does not support that CrM in humans (3–5 grams/day) increases the formation of carcinogenic compounds or cancer risk (primary or metastasis). CrM is likely to be beneficial to help protect and/or recover from the skeletal muscle and body composition issues associated with cancer per se and/or the effects of chemotherapy.
9. CrM does not increase urine production.
10. There is no evidence that CrM adversely affects blood pressure parameters.
11. Animal research suggests that CrM during pregnancy does not negatively impact the mother or offspring. However, there are no well-designed or executed randomized controlled clinical trials on the safety and tolerability of CrM during human pregnancy.
12. In adolescents, CrM can improve measures of sports-specific activities in addition to improving power or sprint speed in adolescents.
13. CrM does not negatively impact male fertility.
14. It is unclear whether the brain requires more CrM than skeletal muscle to increase creatine levels.
15. CrM may positively affect cognition and memory during periods of sleep deprivation in young adults, but not for those with adequate sleep.
16. CrM has the potential to reduce the severity of and/or improve recovery from TBI.

## References

[1] Persky AM, Brazeau GA, Hochhaus G. Pharmacokinetics of the dietary supplement creatine. Clin Pharmacokinet. 2003;42(6):557–41. doi: 10.2165/00003088-200342060-00005 12793840

[2] Harris R, Söderlund K, Hultman E. Elevation of creatine in resting and exercised muscle of normal subjects by creatine supplementation. Clin Sci (Lond) [Internet]. 1992 [cited 2021 Jul 8];83(3):367–374. Available from https://pubmed.ncbi.nlm.nih.gov/1327657/1327657 10.1042/cs0830367

[3] Hultman E, Söderlund K, Timmons J, et al. Muscle creatine loading in men. J Appl Physiol [Internet]. 1996 [cited 2021 Aug 31];81(1):232–237. Available from: https://pubmed.ncbi.nlm.nih.gov/8828669/8828669 10.1152/jappl.1996.81.1.232

[4] Kreider RB, Stout JR. Creatine in health and disease. Nutrients [Internet]. 2021 [cited 2022 Jan 21];13(2):1–28. Available from: https://pubmed.ncbi.nlm.nih.gov/33572884/10.3390/nu13020447 PMC7910963 33572884

[5] Kreider R, Kalman D, Antonio J, et al. International society of sports nutrition position stand: safety and efficacy of creatine supplementation in exercise, sport, and medicine. J Int Soc Sports Nutr [Internet]. 2017 [cited 2021 Jul 8];14(1). Available from. https://pubmed.ncbi.nlm.nih.gov/28615996//10.1186/s12970-017-0173-z PMC5469049 28615996

[6] Jäger R, Purpura M, Shao A, et al. Analysis of the efficacy, safety, and regulatory status of novel forms of creatine. Amino Acids [Internet]. 2011 [cited 2021 Oct 4];40(5):1369. Available from:/pmc/articles/PMC3080578/21424716 10.1007/s00726-011-0874-6 PMC3080578

[7] Kreider RB, Jäger R, Bioavailability PM, et al. Safety, and regulatory status of creatine and related compounds: a critical review. Nutrients [Internet]. 2022 [cited 2022 Dec 17];14(5):1035. Available from: https://pubmed.ncbi.nlm.nih.gov/35268011/35268011 10.3390/nu14051035 PMC8912867

[8] Kreider RB, Kalman DS, Antonio J, et al. International society of sports nutrition position stand: safety and efficacy of creatine supplementation in exercise, sport, and medicine. J Int Soc Sports Nutr [Internet]. 2017 [cited 2023 Jan 4];14(1). Available from: https://pubmed.ncbi.nlm.nih.gov/28615996/10.1186/s12970-017-0173-z PMC5469049 28615996

[9] Ostojic SM, Forbes SC. Perspective: creatine, a conditionally essential nutrient: building the case. Adv Nutr [Internet]. 2022 [cited 2022 Mar 8];13(1):34–37. Available from: https://pubmed.ncbi.nlm.nih.gov/34662902/34662902 10.1093/advances/nmab111 PMC8803499

[10] Antonio J, Candow DG, Forbes SC, et al. Common questions and misconceptions about creatine supplementation: what does the scientific evidence really show? J Int Soc Sports Nutr. 2021;18(1):1–17. doi: 10.1186/s12970-021-00412-w 33557850 PMC7871530

[11] Forbes S, Candow D, Ferreira L, et al. Effects of creatine supplementation on properties of muscle, bone, and brain function in older adults: a narrative review. J Diet Suppl [Internet]. 2021 [cited 2021 Jul 8]. Available from: https://pubmed.ncbi.nlm.nih.gov/33502271/10.1080/19390211.2021.1877232 33502271

[12] Rawson ES, Volek J. Effects of creatine supplementation and resistance training on muscle strength and weightlifting performance. J Strength Cond Res. 2003;17(4):822–831. doi: 10.1519/00124278-200311000-00031 14636102

[13] Lanhers C, Pereira B, Naughton G, et al. Creatine supplementation and upper limb strength performance: a systematic review and meta-analysis. Sports Med [Internet]. 2017 [cited 2022 Mar 3];47(1):163–173. Available from: https://pubmed.ncbi.nlm.nih.gov/27328852/27328852 10.1007/s40279-016-0571-4

[14] Fukuda DH, Smith AE, Kendall KL, et al. The effects of creatine loading and gender on anaerobic running capacity. J Strength Cond Res [Internet]. 2010 [cited 2024 Jun 13];24(7):1826–1833. Available from: https://pubmed.ncbi.nlm.nih.gov/20543729/20543729 10.1519/JSC.0b013e3181e06d0e

[15] Eckerson JM. Creatine as an ergogenic aid for female athletes. Strength Cond J [Internet]. 2016 [cited 2024 Jun 13];38(2):14–23. Available from: https://journals.lww.com/nsca-scj/fulltext/2016/04000/creatine_as_an_ergogenic_aid_for_female_athletes.4.aspx

[16] Wright G, Grandjean P, Pascoe D. The effects of creatine loading on thermoregulation and intermittent sprint exercise performance in a hot humid environment. J Strength Cond Res [Internet]. 2007 [cited 2021 Oct 4];21(3):655–660. Available from: https://pubmed.ncbi.nlm.nih.gov/17685723/17685723 10.1519/R-22186.1

[17] Lanhers C, Pereira B, Naughton G, et al. Creatine supplementation and lower limb strength performance: a systematic review and meta-analyses. Sports Med [Internet]. 2015 [cited 2022 Mar 3];45(9):1285–1294. Available from: https://pubmed.ncbi.nlm.nih.gov/25946994/25946994 10.1007/s40279-015-0337-4

[18] Syrotuik D, Bell G. Acute creatine monohydrate supplementation: a descriptive physiological profile of responders vs. nonresponders. J Strength Cond Res [Internet]. 2004 [cited 2021 Jul 8];18(3):610–617. Available from: https://pubmed.ncbi.nlm.nih.gov/15320650/15320650 10.1519/12392.1

[19] Chilibeck P, Kaviani M, Candow D, et al. Effect of creatine supplementation during resistance training on lean tissue mass and muscular strength in older adults: a meta-analysis. Open Access J Sport Med. 2017;8:213–226. doi: 10.2147/OAJSM.S123529 PMC5679696 29138605

[20] Candow DG, Forbes SC, Chilibeck PD, et al. Variables influencing the effectiveness of creatine supplementation as a therapeutic intervention for Sarcopenia. Front Nutr. 2019;6:124. doi: 10.3389/fnut.2019.00124 31448281 PMC6696725

[21] Brosnan ME, Brosnan JT. The role of dietary creatine. Amino Acids [Internet]. 2016 [cited 2021 Oct 26];48(8):1785–1791. Available from: https://link.springer.com/article/10.1007/s00726-016-2188-1 26874700 10.1007/s00726-016-2188-1

[22] Kaviani M, Shaw K, Chilibeck P. Benefits of creatine supplementation for vegetarians compared to omnivorous athletes: a systematic review. Int J Environ Res Public Health [Internet]. 2020 [cited 2021 Oct 29];17(9):3041. Available from: https://pubmed.ncbi.nlm.nih.gov/32349356/32349356 10.3390/ijerph17093041 PMC7246861

[23] Watt KKO, Garnham AP, Snow RJ. Skeletal muscle total creatine content and creatine transporter gene expression in vegetarians prior to and following creatine supplementation. Int J Sport Nutr Exerc Metab [Internet]. 2004 [cited 2024 Jun 13];14(5):517–531. Available from: https://pubmed.ncbi.nlm.nih.gov/15673098/15673098 10.1123/ijsnem.14.5.517

[24] Moon A, Heywood L, Rutherford S, et al. Creatine supplementation: can it improve quality of life in the elderly without associated resistance training? Curr Aging Sci [Internet]. 2013 [cited 2024 Jun 13];6(3):251–257. Available from: https://pubmed.ncbi.nlm.nih.gov/24304199/24304199 10.2174/1874609806666131204153102

[25] Devries HA, Tichy MW, Housh TJ, et al. A method for estimating physical working capacity at the fatigue threshold (PWCFT). Ergonomics [Internet]. 1987 [cited 2024 Jun 13];30(8):1195–1204. Available from: https://www.tandfonline.com/doi/abs/10.1080/00140138708966008 3691473 10.1080/00140138708966008

[26] Emerson NS, Fukuda DH, Stout JR, et al. Physical working capacity at fatigue threshold (PWCFT) is associated with sarcopenia-related body composition and measures of functionality in older adults. Arch Gerontol Geriatr [Internet]. 2014 [cited 2024 Jun 13];59(2):300–304. Available from: https://pubmed.ncbi.nlm.nih.gov/24856645/24856645 10.1016/j.archger.2014.04.012

[27] Stout J, Sue Graves B, Cramer J, et al. Effects of creatine supplementation on the onset of neuromuscular fatigue threshold and muscle strength in elderly men and women (64–86 years). J Nutr Health Aging [Internet]. 2007 [cited 2021 Jul 20];11:459–464. Available from: https://pubmed.ncbi.nlm.nih.gov/17985060/17985060

[28] DeVries HA, Brodowicz GR, Robertson LD, et al. Estimating physical working capacity and training changes in the elderly at the fatigue threshold (PWCft). Ergonomics [Internet]. 1989 [cited 2024 Jun 13];32(8):967–977. Available from: https://pubmed.ncbi.nlm.nih.gov/2806227/2806227 10.1080/00140138908966858

[29] Sales LP, Pinto AJ, Rodrigues SF, et al. Creatine supplementation (3 g/d) and bone health in older women: a 2-year, randomized, placebo-controlled trial. Journals Gerontol Ser A [Internet]. 2020 [cited 2021 Oct 29];75(5):931–938. Available from: https://academic.oup.com/biomedgerontology/article/75/5/931/5525440 10.1093/gerona/glz162 31257405

[30] Lobo DM, Tritto AC, da Silva LR, et al. Effects of long-term low-dose dietary creatine supplementation in older women. Exp Gerontol. 2015;70:97–104. doi: 10.1016/j.exger.2015.07.012 26192975

[31] Candow DG, Forbes SC, Roberts MD, et al. Creatine O’Clock: does timing of ingestion really influence muscle mass and performance? Front sport act living [Internet]. 2022 [cited 2022 Dec 30];4. Available from https://pubmed.ncbi.nlm.nih.gov/35669557//10.3389/fspor.2022.893714 PMC9163789 35669557

[32] Ribeiro F, Longobardi I, Perim P, et al. Timing of creatine supplementation around exercise: a real concern? Nutrients [Internet]. 2021 [cited 2022 Mar 3];13(8):2844. Available from: https://pubmed.ncbi.nlm.nih.gov/34445003/34445003 10.3390/nu13082844 PMC8401986

[33] Bangsbo J, Hellsten Y. Muscle blood flow and oxygen uptake in recovery from exercise. Acta Physiol Scand [Internet]. 1998 [cited 2024 Jun 13];162(3):305–312. Available from: https://pubmed.ncbi.nlm.nih.gov/9578376/9578376 10.1046/j.1365-201X.1998.0331e.x

[34] Joyner MJ, Casey DP. Regulation of increased blood flow (hyperemia) to muscles during exercise: a hierarchy of competing physiological needs. Physiol Rev [Internet]. 2015 [cited 2022 Mar 3];95(2):549–601. Available from: https://pubmed.ncbi.nlm.nih.gov/25834232/25834232 10.1152/physrev.00035.2013 PMC4551211

[35] Forbes S, Candow D. Timing of creatine supplementation and resistance training: a brief review. J Exerc Nutr [Internet]. 2018 [cited 2021 Oct 6];1:1. Available from: https://www.journalofexerciseandnutrition.com/index.php/JEN/article/view/3337207025

[36] Roberts PA, Fox J, Peirce N, et al. Creatine ingestion augments dietary carbohydrate mediated muscle glycogen supercompensation during the initial 24 h of recovery following prolonged exhaustive exercise in humans. Amino Acids [Internet]. 2016 [cited 2022 Dec 8];48(8):1831–1842. Available from: https://pubmed.ncbi.nlm.nih.gov/27193231/27193231 10.1007/s00726-016-2252-x PMC4974290

[37] Hackett DA, Johnson NA, Chow CM. Training practices and ergogenic aids used by male bodybuilders. J Strength Cond Res [Internet]. 2013 [cited 2022 Mar 3];27(6):1609–1617. Available from: https://pubmed.ncbi.nlm.nih.gov/22990567/22990567 10.1519/JSC.0b013e318271272a

[38] Candow DG, Forbes SC, Roberts MD, et al. Creatine O’Clock: does timing of ingestion really influence muscle mass and performance? Front sport act living [Internet]. 2022 [cited 2023 Apr 19];4. Available from https://pubmed.ncbi.nlm.nih.gov/35669557/10.3389/fspor.2022.893714 PMC9163789 35669557

[39] Green HJ, Chin ER, Ball-Burnett M, et al. Increases in human skeletal muscle Na(+)-k(+)-ATPase concentration with short-term training. Am J Physiol [Internet]. 1993 [cited 2024 Jun 13];264(6):C1538–C1541. Available from: Available from: https://pubmed.ncbi.nlm.nih.gov/8392800/8392800 10.1152/ajpcell.1993.264.6.C1538

[40] Christie DL. Functional insights into the creatine transporter. Subcell Biochem [Internet]. 2007 [cited 2022 Dec 18];46:99–118. Available from: https://pubmed.ncbi.nlm.nih.gov/18652074/18652074 10.1007/978-1-4020-6486-9_6

[41] Cribb PJ, Hayes A. Effects of supplement timing and resistance exercise on skeletal muscle hypertrophy. Med Sci Sports Exerc [Internet]. 2006 [cited 2022 Mar 8];38(11):1918–1925. Available from: https://pubmed.ncbi.nlm.nih.gov/17095924/17095924 10.1249/01.mss.0000233790.08788.3e

[42] Forbes SC, Krentz JR, Candow DG. Timing of creatine supplementation does not influence gains in unilateral muscle hypertrophy or strength from resistance training in young adults: a within-subject design. J Sports Med Phys Fitness [Internet]. 2021 [cited 2022 Mar 8];61(9):1219–1225. Available from: https://pubmed.ncbi.nlm.nih.gov/34610729/34610729 10.23736/S0022-4707.20.11668-2

[43] Candow DG, Zello GA, Ling B, et al. Comparison of creatine supplementation before versus after supervised resistance training in healthy older adults. Res Sport Med [Internet]. 2014 [cited 2022 Mar 8];22(1):61–74. Available from: https://pubmed.ncbi.nlm.nih.gov/24392772/10.1080/15438627.2013.852088 24392772

[44] Antonio J, Ciccone V. The effects of pre versus post workout supplementation of creatine monohydrate on body composition and strength. J Int Soc Sports Nutr [Internet]. 2013 [cited 2022 Mar 8];10(1). Available from: https://pubmed.ncbi.nlm.nih.gov/23919405/10.1186/1550-2783-10-36 PMC3750511 23919405

[45] Jurado-Castro JM, Campos-Pérez J, Vilches-Redondo MÁ, et al. Morning versus evening intake of creatine in elite female handball players. Int J Environ Res Public Health [Internet]. 2021 [cited 2022 Mar 8];19(1):393. Available from: https://pubmed.ncbi.nlm.nih.gov/35010653/35010653 10.3390/ijerph19010393 PMC8744932

[46] Dinan NE, Hagele AM, Jagim AR, et al. Effects of creatine monohydrate timing on resistance training adaptations and body composition after 8 weeks in male and female collegiate athletes. Front Sport Act Living [Internet]. 2022 [cited 2024 Jun 13];4. Available from: https://pubmed.ncbi.nlm.nih.gov/36465581/10.3389/fspor.2022.1033842 PMC9708881 36465581

[47] Candow D, Vogt E, Johannsmeyer S, et al. Strategic creatine supplementation and resistance training in healthy older adults. Appl Physiol Nutr Metab [Internet]. 2015 [cited 2021 Jul 7];40(7):689–694. Available from: https://pubmed.ncbi.nlm.nih.gov/25993883/25993883 10.1139/apnm-2014-0498

[48] Kerksick CM, Wilborn CD, Roberts MD, et al. ISSN exercise & sports nutrition review update: research & recommendations. J Int Soc Sports Nutr [Internet]. 2018 [cited 2024 Jun 13];15(1). Available from: https://pubmed.ncbi.nlm.nih.gov/30068354/10.1186/s12970-018-0242-y PMC6090881 30068354

[49] Steenge GR, Simpson EJ, Greenhaff PL. Protein- and carbohydrate-induced augmentation of whole body creatine retention in humans. J Appl Physiol [Internet]. 2000 [cited 2022 Jan 20];89(3):1165–1171. Available from: https://pubmed.ncbi.nlm.nih.gov/10956365/10956365 10.1152/jappl.2000.89.3.1165

[50] Rooney K, Bryson J, Phuyal J, et al. Creatine supplementation alters insulin secretion and glucose homeostasis in vivo. Metabolism [Internet]. 2002 [cited 2024 Jun 13];51(4):518–522. Available from: https://pubmed.ncbi.nlm.nih.gov/11912564/11912564 10.1053/meta.2002.31330

[51] Gualano B, Salles Painneli DE, Roschel VH, et al. Creatine in type 2 diabetes: a randomized, double-blind, placebo-controlled trial. Med Sci Sports Exerc [Internet]. 2011 [cited 2021 Jul 19];43(5):770–778. Available from: https://pubmed.ncbi.nlm.nih.gov/20881878/20881878 10.1249/MSS.0b013e3181fcee7d

[52] Alves CRR, Ferreira JC, De Siqueira-Filho MA, et al. Creatine-induced glucose uptake in type 2 diabetes: a role for AMPK-α? Amino Acids [Internet]. 2012 [cited 2024 Jun 13];43(4):1803–1807. Available from: https://pubmed.ncbi.nlm.nih.gov/22349765/22349765 10.1007/s00726-012-1246-6

[53] Eijnde O’, Ursø B, Richter BEA, et al. Effect of oral creatine supplementation on human muscle GLUT4 protein content after immobilization. Diabetes [Internet]. 2001 [cited 2024 Jun 13];50(1):18–23. Available from: https://pubmed.ncbi.nlm.nih.gov/11147785/11147785 10.2337/diabetes.50.1.18

[54] Green A, Hultman E, Macdonald I, et al. Carbohydrate ingestion augments skeletal muscle creatine accumulation during creatine supplementation in humans. Am J Physiol [Internet]. 1996 [cited 2021 Jul 8];271(5):E821–E826. Available from: https://pubmed.ncbi.nlm.nih.gov/8944667/8944667 10.1152/ajpendo.1996.271.5.E821

[55] Nelson AG, Arnall DA, Kokkonen J, et al. Muscle glycogen supercompensation is enhanced by prior creatine supplementation. Med Sci Sports Exerc [Internet]. 2001 [cited 2024 Jun 13];33:1096–1100. Available from: https://pubmed.ncbi.nlm.nih.gov/11445755/11445755 10.1097/00005768-200107000-00005

[56] Burke DG, Chilibeck PD, Parise G, et al. Effect of α-Lipoic acid combined with creatine monohydrate on human skeletal muscle creatine and phosphagen concentration. Int J Sport Nutr Exerc Metab [Internet]. 2003 [cited 2022 Jan 20];13(3):294–302. Available from: https://pubmed.ncbi.nlm.nih.gov/14669930/14669930 10.1123/ijsnem.13.3.294

[57] Cribb PJ, Williams AD, Stathis CG, et al. Effects of whey isolate, creatine, and resistance training on muscle hypertrophy. Med Sci Sports Exerc [Internet]. 2007 [cited 2024 Jun 13];39(2):298–307. Available from: https://pubmed.ncbi.nlm.nih.gov/17277594/17277594 10.1249/01.mss.0000247002.32589.ef

[58] Cornish SM, Candow DG, Jantz NT, et al. Conjugated linoleic acid combined with creatine monohydrate and whey protein supplementation during strength training. Int J Sport Nutr Exerc Metab. 2009;19(1):79–96. doi: 10.1123/ijsnem.19.1.79 19403955

[59] O’Connor DM, Crowe MJ. EFFECTS of SIX WEEKS of β-hydroxy-β-methylbutyrate (HMB) and HMB/CREATINE SUPPLEMENTATION on STRENGTH, POWER, and ANTHROPOMETRY of HIGHLY TRAINED ATHLETES. J Strength Cond Res [Internet]. 2007 [cited 2024 Jun 13];21(2):419–423. Available from: https://pubmed.ncbi.nlm.nih.gov/17530933/17530933 10.1519/R-15974.1

[60] Crowe MJ, Dm O, Lukins JE. The effects of ß-hydroxy-ß-methylbutyrate (HMB) and HMB/Creatine supplementation on indices of health in highly trained athletes. Int J Sport Nutr Exerc Metab [Internet]. 2003 [cited 2024 Jun 13];13(2):184–197. Available from: https://pubmed.ncbi.nlm.nih.gov/12945829/12945829 10.1123/ijsnem.13.2.184

[61] Mangine GT, Vandusseldorp TA, Hester GM, et al. The addition of β-Hydroxy β-Methylbutyrate (HMB) to creatine monohydrate supplementation does not improve anthropometric and performance maintenance across a collegiate rugby season. [cited 2024 Jun 13]. doi: 10.1186/s12970-020-00359-4 PMC7254750 32460801

[62] Fernández-Landa J, Fernández-Lázaro D, Calleja-González J, et al. Effect of Ten weeks of creatine monohydrate plus HMB supplementation on athletic performance tests in elite male endurance athletes. Nutrients [Internet]. 2020 [cited 2024 Jun 13];12. Available from 193. Available from: https://pubmed.ncbi.nlm.nih.gov/31936727/31936727 10.3390/nu12010193 PMC7019716

[63] Jówko E, Ostaszewski P, Jank M, et al. Creatine and β-hydroxy-β-methylbutyrate (HMB) additively increase lean body mass and muscle strength during a weight-training program. Nutrition [Internet]. 2001 [cited 2024 Jun 13];17(7–8):558–566. Available from: https://pubmed.ncbi.nlm.nih.gov/11448573/11448573 10.1016/s0899-9007(01)00540-8

[64] Barber JJ, Mcdermott AY, Mcgaughey KJ, et al. Effects of combined creatine and sodium bicarbonate supplementation on repeated sprint performance in trained men. J Strength Cond Res [Internet]. 2013 [cited 2024 Jun 13];27(1):252–258. Available from: https://pubmed.ncbi.nlm.nih.gov/23254493/23254493 10.1519/JSC.0b013e318252f6b7

[65] Kim J. Effects of combined creatine and sodium bicarbonate supplementation on soccer-specific performance in elite soccer players: a randomized controlled trial. Int J Environ Res Public Health [Internet]. 2021 [cited 2024 Jun 13];18(13):6919. Available from: https://pubmed.ncbi.nlm.nih.gov/34203286/34203286 10.3390/ijerph18136919 PMC8297001

[66] Mero AA, Keskinen KL, Malvela MT, et al. Combined creatine and sodium bicarbonate supplementation enhances interval swimming. J Strength Cond Res [Internet]. 2004 [cited 2024 Jun 13];18(2):306–310. Available from: https://pubmed.ncbi.nlm.nih.gov/15142001/15142001 10.1519/R-12912.1

[67] Sarshin A, Fallahi V, Forbes SC, et al. Short-term co-ingestion of creatine and sodium bicarbonate improves anaerobic performance in trained taekwondo athletes. J Int Soc Sports Nutr [Internet]. 2021 [cited 2024 Jun 13];18(1). Available from: https://pubmed.ncbi.nlm.nih.gov/33478522/10.1186/s12970-021-00407-7 PMC7819230 33478522

[68] Samadi M, Askarian A, Shirvani H, et al. Effects of four weeks of Beta-alanine supplementation combined with one week of creatine loading on physical and cognitive performance in military personnel. Int J Environ Res Public Health [Internet]. 2022 [cited 2023 Apr 19];19(13):7992. Available from: https://pubmed.ncbi.nlm.nih.gov/35805647/35805647 10.3390/ijerph19137992 PMC9265371

[69] Hoffman J, Ratamess N, Kang J, et al. Effect of creatine and ß-alanine supplementation on performance and endocrine responses in strength/power athletes. Int J Sport Nutr Exerc Metab [Internet]. 2006 [cited 2023 May 24];16(4):430–446. Available from: https://pubmed.ncbi.nlm.nih.gov/17136944/17136944 10.1123/ijsnem.16.4.430

[70] Stout JR, Cramer JT, Mielke M, et al. Effects of twenty-eight days of beta-alanine and creatine monohydrate supplementation on the physical working capacity at neuromuscular fatigue threshold. J Strength Cond Res [Internet]. 2006 [cited 2024 Jun 13];20(4):928–931. Available from: https://pubmed.ncbi.nlm.nih.gov/17194255/17194255 10.1519/R-19655.1

[71] Zoeller RF, Stout JR, O’Kroy JA, et al. Effects of 28 days of beta-alanine and creatine monohydrate supplementation on aerobic power, ventilatory and lactate thresholds, and time to exhaustion. Amino Acids [Internet]. 2007 [cited 2024 Jun 13];33(3):505–510. Available from: https://pubmed.ncbi.nlm.nih.gov/16953366/16953366 10.1007/s00726-006-0399-6

[72] Kerksick CM, Wilborn CD, Campbell WI, et al. The effects of creatine monohydrate supplementation with and without D-pinitol on resistance training adaptations. J Strength Cond Res [Internet]. 2009 [cited 2024 Jun 13];23(9):2673–2682. Available from: https://pubmed.ncbi.nlm.nih.gov/19858753/19858753 10.1519/JSC.0b013e3181b3e0de

[73] Taylor L, Poole C, Pena E, et al. Effects of combined creatine plus fenugreek extract vs. Creatine plus carbohydrate supplementation on resistance training adaptations. J Sports Sci Med [Internet]. 2011 [cited 2024 Jun 13];10:254. Available from:/pmc/articles/PMC3761853/24149869 PMC3761853

[74] Islam H, Yorgason NJ, Hazell TJ. Creatine co-ingestion with carbohydrate or cinnamon extract provides no added benefit to anaerobic performance. Eur J Sport Sci [Internet]. 2016 [cited 2024 Jun 13];16(6):685–693. Available from: https://pubmed.ncbi.nlm.nih.gov/26313717/26313717 10.1080/17461391.2015.1071877

[75] Oliver JM, Jagim AR, Pischel I, et al. Effects of short-term ingestion of Russian tarragon prior to creatine monohydrate supplementation on whole body and muscle creatine retention and anaerobic sprint capacity: a preliminary investigation. J Int Soc Sports Nutr [Internet]. 2014 [cited 2024 Jun 13];11(1). Available from: https://pubmed.ncbi.nlm.nih.gov/24568653/10.1186/1550-2783-11-6 PMC3975968 24568653

[76] Polyviou TP, Easton C, Beis L, et al. Effects of glycerol and creatine hyperhydration on doping-relevant blood parameters. Nutrients [Internet]. 2012 [cited 2021 Oct 4];4(9):1171. Available from:/pmc/articles/PMC3475229/23112907 10.3390/nu4091171 PMC3475229

[77] Easton C, Turner S, Pitsiladis YP. Creatine and glycerol hyperhydration in trained subjects before exercise in the heat. Int J Sport Nutr Exerc Metab [Internet]. 2007 [cited 2024 Jun 13];17(1):70–91. Available from: https://pubmed.ncbi.nlm.nih.gov/17460334/17460334 10.1123/ijsnem.17.1.70

[78] Beis LY, Polyviou T, Malkova D, et al. The effects of creatine and glycerol hyperhydration on running economy in well trained endurance runners. J Int Soc Sports Nutr [Internet]. 2011 [cited 2024 Jun 13];8(1). Available from: https://pubmed.ncbi.nlm.nih.gov/22176668/10.1186/1550-2783-8-24 PMC3283512 22176668

[79] Beis LY, Polyviou T, Malkova D, et al. The effects of creatine and glycerol hyperhydration on running economy in well trained endurance runners. J Int Soc Sports Nutr [Internet]. 2011 [cited 2021 Oct 4];8(1):24. Available from: http://www.jissn.com/content/8/1/24/22176668 10.1186/1550-2783-8-24 PMC3283512

[80] Greenhaff PL, Bodin K, Soderlund K, et al. Effect of oral creatine supplementation on skeletal muscle phosphocreatine resynthesis. Am J Physiol [Internet]. 1994 [cited 2024 Mar 19];266(5):E725–E730. Available from: https://pubmed.ncbi.nlm.nih.gov/8203511/8203511 10.1152/ajpendo.1994.266.5.E725

[81] Vanakoski J, Kosunen V, Meririnne E, et al. Creatine and caffeine in anaerobic and aerobic exercise: effects on physical performance and pharmacokinetic considerations. Int J Clin Pharmacol Ther. 1998;36(5):258–262. Available from: http://www.jissn.com/content/8/1/24 9629989

[82] Tarnopolsky MA. Effect of caffeine on the neuromuscular system — potential as an ergogenic aid. Appl Physiol Nutr Metab [Internet]. 2008 [cited 2024 Oct 31];33(6):1284–1289. Available from: https://pubmed.ncbi.nlm.nih.gov/19088790/19088790 10.1139/H08-121

[83] Vandenberghe K, Gillis N, Van Leemputte M, et al. Caffeine counteracts the ergogenic action of muscle creatine loading. J Appl Physiol [Internet]. 1996 [cited 2022 Apr 8];80(2):452–457. Available from: https://pubmed.ncbi.nlm.nih.gov/8929583/8929583 10.1152/jappl.1996.80.2.452

[84] Hespel P, ’Eijnde TBO, Van LM. Opposite actions of caffeine and creatine on muscle relaxation time in humans. J Appl Physiol [Internet]. 2002 [cited 2022 Apr 8];92(2):513–518. Available from: https://pubmed.ncbi.nlm.nih.gov/11796658/11796658 10.1152/japplphysiol.00255.2001

[85] Trexler ET, Smith-Ryan AE, Roelofs EJ, et al. Effects of coffee and caffeine anhydrous intake during creatine loading. J Strength Cond Res [Internet]. 2016 [cited 2024 Jun 13];30(5):1438–1446. Available from: https://pubmed.ncbi.nlm.nih.gov/26439785/26439785 10.1519/JSC.0000000000001223 PMC4808512

[86] Marinho AH, Gonçalves JS, Araújo PK, et al. Effects of creatine and caffeine ingestion in combination on exercise performance: a systematic review. Crit Rev Food Sci Nutr [Internet]. 2023 [cited 2024 Jun 13];63(20):4785–4798. Available from: https://pubmed.ncbi.nlm.nih.gov/34845944/34845944 10.1080/10408398.2021.2007470

[87] Elosegui S, López-Seoane J, Martínez-Ferrán M, et al. Interaction between caffeine and creatine when used as concurrent ergogenic supplements: a systematic review. Int J Sport Nutr Exerc Metab [Internet]. 2022 [cited 2024 Jun 13];32(4):285–295. Available from: https://pubmed.ncbi.nlm.nih.gov/35016154/35016154 10.1123/ijsnem.2021-0262

[88] Doherty M, Smith PM, Davison RCR, et al. Caffeine is ergogenic after supplementation of oral creatine monohydrate. Med Sci Sports Exerc [Internet]. 2002 [cited 2024 Jun 13];34(11):1785–1792. Available from: https://pubmed.ncbi.nlm.nih.gov/12439084/12439084 10.1097/00005768-200211000-00015

[89] Lee CL, Lin JC, Cheng CF. Effect of caffeine ingestion after creatine supplementation on intermittent high-intensity sprint performance. Eur J Appl Physiol [Internet]. 2011 [cited 2024 Jun 13];111(8):1669–1677. Available from: https://pubmed.ncbi.nlm.nih.gov/21207054/21207054 10.1007/s00421-010-1792-0

[90] Orange S, Sadler I. The effect of caffeine ingestion after creatine supplementation on peak torque production. Grad J Sport Exerc Phys Educ Res. 2015;3:80–92. Available from: https://pubmed.ncbi.nlm.nih.gov/12439084//

[91] Pakulak A, Candow DG, Totosy de Zepetnek J, et al. Effects of creatine and caffeine supplementation during resistance training on body composition, strength, endurance, rating of perceived exertion and fatigue in trained young adults. J Diet Suppl [Internet]. 2021;19(5):587–602. Available from doi:10.1080/19390211.2021.1904085 33759701

[92] Jeronimo D, Germano M, Fiorante F, et al. Caffeine potentiates the ergogenic effects of creatine. J Exerc Physiol Online. 2017:20.

[93] Burke R, Piñero A, Coleman M, et al. The effects of creatine supplementation combined with resistance training on regional measures of muscle hypertrophy: a systematic review with meta-analysis. Nutrients [Internet]. 2023 [cited 2023 Apr 29];15(9):2116. Available from: https://www.mdpi.com/2072-6643/15/9/2116/htm 37432300 10.3390/nu15092116 PMC10180745

[94] Forbes SC, Candow DG. Creatine and strength training in older adults: an update. Transl Exerc Biomed [Internet]. 2024 [cited 2024 Oct 28]. p. 1–11. Available from: https://www.degruyter.com/document/doi/10.1515/teb-2024-0019/html

[95] Louis M, Poortmans JR, Francaux M, et al. Creatine supplementation has no effect on human muscle protein turnover at rest in the postabsorptive or fed states. Am J Physiol Endocrinol Metab [Internet]. 2003 [cited 2024 Jun 13];284(4):E764–E770. Available from: https://pubmed.ncbi.nlm.nih.gov/12475751/12475751 10.1152/ajpendo.00338.2002

[96] Louis M, Poortmans JR, Francaux M, et al. No effect of creatine supplementation on human myofibrillar and sarcoplasmic protein synthesis after resistance exercise. Am J Physiol Endocrinol Metab [Internet]. 2003 [cited 2024 Jun 13];285(5):E1089–E1094. Available from: https://pubmed.ncbi.nlm.nih.gov/12824083/12824083 10.1152/ajpendo.00195.2003

[97] Parise G, Mihic S, MacLennan D, et al. Effects of acute creatine monohydrate supplementation on leucine kinetics and mixed-muscle protein synthesis. J Appl Physiol [Internet]. 2001 [cited 2024 Jun 13];91(3):1041–1047. Available from: https://pubmed.ncbi.nlm.nih.gov/11509496/11509496 10.1152/jappl.2001.91.3.1041

[98] Candow DG, Little JP, Chilibeck PD, et al. Low-dose creatine combined with protein during resistance training in older men. Med Sci Sports Exerc [Internet]. 2008 [cited 2021 Jul 19];40(9):1645–1652. Available from: https://journals.lww.com/acsm-msse/Fulltext/2008/09000/Low_Dose_Creatine_Combined_with_Protein_during.13.aspx 18685526 10.1249/MSS.0b013e318176b310

[99] Johannsmeyer S, Candow DG, Brahms CM, et al. Effect of creatine supplementation and drop-set resistance training in untrained aging adults. Exp Gerontol. 2016;83:112–119. doi: 10.1016/j.exger.2016.08.005 27523919

[100] Cordingley DM, Cornish SM, Candow DG. Anti-inflammatory and anti-catabolic effects of creatine supplementation: a brief review. Nutrients [Internet]. 2022 [cited 2022 Dec 8];14(3):544. Available from: https://pubmed.ncbi.nlm.nih.gov/35276903/35276903 10.3390/nu14030544 PMC8839648

[101] Shaw SK, Ma S, Kim MB, et al. Coordinated redistribution of leukocyte LFA-1 and endothelial cell ICAM-1 accompany neutrophil transmigration. J Exp Med [Internet]. 2004 [cited 2024 Jun 13];200(12):1571–1580. Available from: https://pubmed.ncbi.nlm.nih.gov/15611287/15611287 10.1084/jem.20040965 PMC2212000

[102] Nosaka K, Newton M. Concentric or eccentric training effect on eccentric exercise-induced muscle damage. Med Sci Sports Exerc [Internet]. 2002 [cited 2024 Jun 13];34(1):63–69. Available from: https://pubmed.ncbi.nlm.nih.gov/11782649/11782649 10.1097/00005768-200201000-00011

[103] Smith LL, Wells JM, Houmard JA, et al. Increases in plasma prostaglandin E 2 after eccentric exercise [Internet]. 1993 [cited 2024 Jun 13];25(8):451–452. Available from: https://pubmed.ncbi.nlm.nih.gov/8225195/10.1055/s-2007-1002145 8225195

[104] Santos RVT, Bassit RA, Caperuto EC, et al. The effect of creatine supplementation upon inflammatory and muscle soreness markers after a 30km race. Life Sci. 2004;75(16):1917–1924. doi: 10.1016/j.lfs.2003.11.036 15306159

[105] Bassit RA, Curi R, Rosa Lfbp C. Creatine supplementation reduces plasma levels of pro-inflammatory cytokines and PGE2 after a half-ironman competition. Amin Acids 2007 352 [Internet]. 2007 [cited 2021 Oct 4];35(2):425–431. Available from: https://link.springer.com/article/10.1007/s00726-007-0582-4 10.1007/s00726-007-0582-4 17917696

[106] Deminice R, Rosa FT, Franco GS, et al. Effects of creatine supplementation on oxidative stress and inflammatory markers after repeated-sprint exercise in humans. Nutrition. 2013;29(9):1127–1132. doi: 10.1016/j.nut.2013.03.003 23800565

[107] Rawson ES, Conti MP, Miles MP. Creatine supplementation does not reduce muscle damage or enhance recovery from resistance exercise. J Strength Cond Res [Internet]. 2007 [cited 2024 Jun 13];21(4):1208–1213. Available from: https://pubmed.ncbi.nlm.nih.gov/18076246/18076246 10.1519/R-21076.1

[108] Gualano B, Artioli GG, Poortmans JR, et al. Exploring the therapeutic role of creatine supplementation. Amino Acids [Internet]. 2010 [cited 2022 Jan 20];38(1):31–44. Available from: https://pubmed.ncbi.nlm.nih.gov/19253023/19253023 10.1007/s00726-009-0263-6

[109] Gualano B, Roschel H, Lancha AH, et al. In sickness and in health: the widespread application of creatine supplementation. Amino Acids. 2012;43(2):519–529. doi: 10.1007/s00726-011-1132-7 22101980

[110] Hausmann O, Fouad K, Wallimann T, et al. Protective effects of oral creatine supplementation on spinal cord injury in rats. Spinal Cord [Internet]. 2002 [cited 2021 Oct 25];40(9):449–456. Available from: https://www.nature.com/articles/3101330 12185606 10.1038/sj.sc.3101330

[111] Özkan Ö, Duman Ö, Haspolat Ş, et al. Effect of systemic creatine monohydrate supplementation on denervated muscle during reinnervation: experimental study in the rat. J Reconstr Microsurg [Internet]. 2005 [cited 2024 Jun 13];21(8):573–580. Available from: https://pubmed.ncbi.nlm.nih.gov/16292735/16292735 10.1055/s-2005-922438

[112] Hespel P, Eijnde BOT, Van Leemputte M, et al. Oral creatine supplementation facilitates the rehabilitation of disuse atrophy and alters the expression of muscle myogenic factors in humans. J Physiol [Internet]. 2001 [cited 2024 Jun 13];536(2):625–633. Available from: https://pubmed.ncbi.nlm.nih.gov/11600695/11600695 10.1111/j.1469-7793.2001.0625c.xd PMC2278864

[113] Johnston APW, Burke DG, Macneil LG, et al. Effect of creatine supplementation during cast-induced immobilization on the preservation of muscle mass, strength, and endurance. J Strength Cond Res [Internet]. 2009 [cited 2024 Jun 13];23(1):116–120. Available from: https://pubmed.ncbi.nlm.nih.gov/19130643/19130643 10.1519/jsc.0b013e31818efbcc

[114] Backx EMP, Hangelbroek R, Snijders T, et al. Creatine loading does not preserve muscle mass or strength during leg immobilization in healthy, young males: a randomized controlled trial. Sports Med [Internet]. 2017 [cited 2024 Jun 13];47(8):1661–1671. Available from: https://pubmed.ncbi.nlm.nih.gov/28054322/28054322 10.1007/s40279-016-0670-2 PMC5507980

[115] Tyler TF, Nicholas SJ, Hershman EB, et al. The effect of creatine supplementation on strength recovery after anterior cruciate ligament (ACL) reconstruction: a randomized, placebo-controlled, double-blind trial. Am J Sports Med [Internet]. 2004 [cited 2024 Jun 13];32(2):383–388. Available from: https://pubmed.ncbi.nlm.nih.gov/14977662/14977662 10.1177/0363546503261731

[116] Wyss M, Kaddurah-Daouk R. Creatine and creatinine metabolism. Physiol Rev. 2000;80(3):1107–1213. doi: 10.1152/physrev.2000.80.3.1107 10893433

[117] Mirvish SS, Huang Q, Chen SC, et al. Metabolism of carcinogenic nitrosamines in the rat and human esophagus and induction of esophageal adenocarcinoma in rats. Endoscopy [Internet]. 1993 [cited 2024 Jun 14];25(9):627–631. Available from: https://pubmed.ncbi.nlm.nih.gov/8119218/8119218 10.1055/s-2007-1010418

[118] Mirvish SS, Reimers KJ, Kutler B, et al. Nitrate and nitrite concentrations in human saliva for men and women at different ages and times of the day and their consistency over time. Eur J Cancer Prev [Internet]. 2000 [cited 2024 Jun 14];9(5):335–342. Available from: https://pubmed.ncbi.nlm.nih.gov/11075887/11075887 10.1097/00008469-200010000-00008

[119] Mirvish S. The etiology of gastric cancer. Intragastric nitrosamide formation and other theories. J Natl Cancer Inst. 1983;71(3):629–647. 6350677

[120] Hartman PE. Review: putative mutagens and carcinogens in foods. I. Nitrate/nitrite ingestion and gastric cancer mortality. Environ Mutagen [Internet]. 1983 [cited 2024 Jun 26];5(1):111–121. Available from: https://pubmed.ncbi.nlm.nih.gov/6832084/6832084 10.1002/em.2860050112

[121] Pfau W, Rosenvold K, Young JF. Formation of mutagenic heterocyclic aromatic amines in fried pork from duroc and landrace pigs upon feed supplementation with creatine monohydrate. Food Chem Toxicol [Internet]. 2006 [cited 2024 Jun 14];44(12):2086–2091. Available from: https://pubmed.ncbi.nlm.nih.gov/16973250/16973250 10.1016/j.fct.2006.07.011

[122] dos S PR, Dörr FA, Pinto E, et al. Can creatine supplementation form carcinogenic heterocyclic amines in humans? J Physiol [Internet]. 2015 [cited 2021 Oct 26];593(17):3959–3971. Available from:/pmc/articles/PMC4575580/26148133 10.1113/JP270861 PMC4575580

[123] Ostojic SM, Grasaas E, Cvejic J. Dietary creatine and cancer risk in the U.S. population: NHANES 2017–2020. J Funct Foods. 2023;108:105733. doi: 10.1016/j.jff.2023.105733

[124] Zhang L, Zhu Z, Yan H, et al. Creatine promotes cancer metastasis through activation of Smad2/3. Cell Metab. 2021;33(6):1111–1123.e4. doi: 10.1016/j.cmet.2021.03.009 33811821

[125] Tarnopolsky M, Bourgeois J, Snow R, et al. Histological assessment of intermediate- and long-term creatine monohydrate supplementation in mice and rats. Am J Physiol Regul Integr Comp Physiol [Internet]. 2003 [cited 2021 Oct 26];285(4):R762–R769. Available from: https://pubmed.ncbi.nlm.nih.gov/12959920/12959920 10.1152/ajpregu.00270.2003

[126] Sun W, Liu R, Gao X, et al. Targeting serine-glycine-one-carbon metabolism as a vulnerability in cancers. Biomark Res [Internet]. 2023 [cited 2024 Jun 14];11(1). Available from: https://pubmed.ncbi.nlm.nih.gov/37147729/10.1186/s40364-023-00487-4 PMC10161514 37147729

[127] Baggetto L, Clottes E, Vial C. Low mitochondrial proton leak due to high membrane cholesterol content and cytosolic creatine kinase as two features of the deviant bioenergetics of Ehrlich and AS30-D tumor cells. Cancer Res. 1992;52(18):4935–4941. 1516050

[128] Mi Y, Li Q, Liu B, et al. Ubiquitous mitochondrial creatine kinase promotes the progression of gastric cancer through a JNK-MAPK/JUN/HK2 axis regulated glycolysis. Gastric Cancer [Internet]. 2023 [cited 2024 Jun 14];26(1):69–81. Available from: https://pubmed.ncbi.nlm.nih.gov/36114400/36114400 10.1007/s10120-022-01340-7 PMC9813075

[129] Ayyappan V, Jenkinson NM, Tressler CM, et al. Context-dependent roles for ubiquitous mitochondrial creatine kinase CKMT1 in breast cancer progression. Cell Rep [Internet]. 2024 [cited 2024 Jun 14];43(4):114121. Available from: https://pubmed.ncbi.nlm.nih.gov/38615320/38615320 10.1016/j.celrep.2024.114121 PMC11100297

[130] Patel R, Ford CA, Rodgers L, et al. Cyclocreatine suppresses creatine metabolism and impairs prostate cancer progression. Cancer Res [Internet]. 2022 [cited 2024 Jun 14];82(14):2565–2575. Available from: https://pubmed.ncbi.nlm.nih.gov/35675421/35675421 10.1158/0008-5472.CAN-21-1301 PMC9381098

[131] Lillie J, O’Keefe M, Valinski H, et al. Cyclocreatine (1-carboxymethyl-2-iminoimidazolidine) inhibits growth of a broad spectrum of cancer cells derived from solid tumors. Cancer Res. 1993;53(13):3172–3178. 8319226

[132] Cella PS, Marinello PC, Padilha CS, et al. Creatine supplementation does not promote tumor growth or enhance tumor aggressiveness in walker-256 tumor-bearing rats. Nutrition [Internet]. 2020 [cited 2024 Jun 14]. p. 79–80. Available from: https://pubmed.ncbi.nlm.nih.gov/32882636/10.1016/j.nut.2020.110958 32882636

[133] Di BS, Ma X, Wang X, et al. Creatine uptake regulates CD8 T cell antitumor immunity. J Exp Med [Internet]. 2019 [cited 2021 Oct 26];216(12):2869–2882. Available from:/pmc/articles/PMC6888972/31628186 10.1084/jem.20182044 PMC6888972

[134] Miller EE, Evans AE, Cohn M. Inhibition of rate of tumor growth by creatine and cyclocreatine. Proc Natl Acad Sci U S A [Internet]. 1993 [cited 2024 Jun 14];90(8):3304–3308. Available from: https://pubmed.ncbi.nlm.nih.gov/8475072/8475072 10.1073/pnas.90.8.3304 PMC46288

[135] Kristensen CA, Askenasy N, Jain RK, et al. Creatine and cyclocreatine treatment of human colon adenocarcinoma xenografts: 31P and 1H magnetic resonance spectroscopic studies. Br J Cancer [Internet]. 1999 [cited 2024 Jun 14];79(2):278–285. Available from: https://pubmed.ncbi.nlm.nih.gov/9888469/9888469 10.1038/sj.bjc.6690045 PMC2362210

[136] Li B, Yang L. Creatine in T cell antitumor immunity and cancer immunotherapy. Nutrients [Internet]. 2021 [cited 2024 Jun 14];13(5):1633. Available from: https://pubmed.ncbi.nlm.nih.gov/34067957/34067957 10.3390/nu13051633 PMC8152274

[137] Brose A, Parise G, Tarnopolsky M. Creatine supplementation enhances isometric strength and body composition improvements following strength exercise training in older adults. J Gerontol A Biol Sci Med Sci [Internet]. 2003 [cited 2021 Oct 29];58(1):B11–B19. Available from: https://pubmed.ncbi.nlm.nih.gov/12560406/10.1093/gerona/58.1.b11 12560406

[138] Chrusch M, Chilibeck P, Chad K, et al. Creatine supplementation combined with resistance training in older men. Med Sci Sports Exerc [Internet]. 2001 [cited 2021 Oct 6];33(12):2111–2117. Available from: https://journals.lww.com/acsm-msse/Fulltext/2001/12000/Creatine_supplementation_combined_with_resistance.21.aspx 11740307 10.1097/00005768-200112000-00021

[139] Neves M, Gualano B, Roschel H, et al. Beneficial effect of creatine supplementation in knee osteoarthritis. Med Sci Sports Exerc [Internet]. 2011 [cited 2021 Oct 4];43(8):1538–1543. Available from: https://journals.lww.com/acsm-msse/Fulltext/2011/08000/Beneficial_Effect_of_Creatine_Supplementation_in.20.aspx 21311365 10.1249/MSS.0b013e3182118592

[140] Forbes SC, Candow DG, Ostojic SM, et al. Meta-analysis examining the importance of creatine ingestion strategies on lean tissue mass and strength in older adults. 2021;13(6):1912. doi: 10.3390/nu13061912 PMC8229907 34199420

[141] Coletta AM, Simon LH, Maslana K, et al. Creatine supplementation and resistance training to preserve muscle mass and attenuate cancer progression (CREATINE-52): a protocol for a double-blind randomized controlled trial. BMC Cancer [Internet]. 2024 [cited 2024 Jun 14];24(1). Available from: https://pubmed.ncbi.nlm.nih.gov/38637770/10.1186/s12885-024-12260-3 PMC11025211 38637770

[142] Patel DI, Gonzalez A, Moon C, et al. Exercise and creatine supplementation to augment the adaptation of exercise training among breast cancer survivors completing chemotherapy: protocol for an open-label randomized controlled trial (the THRIVE study). JMIR Res Protoc [Internet]. 2022 [cited 2024 Jun 14];11(4):e26827. Available from: https://pubmed.ncbi.nlm.nih.gov/35363152/35363152 10.2196/26827 PMC9015753

[143] Martinez Aguirre-Betolaza A, Cacicedo J, Castañeda-Babarro A. Creatine supplementation and resistance training in patients with breast cancer (CaRtiC study): protocol for a randomized controlled trial. Am J Clin Oncol [Internet]. 2024 [cited 2024 Jun 14];47(4):161–168. Available from: https://pubmed.ncbi.nlm.nih.gov/38018533/38018533 10.1097/COC.0000000000001070

[144] Bredahl EC, Busekrus RB, Hydock DS. The combined effect of creatine and resistance training on doxorubicin-induced muscle dysfunction. Nutr Cancer [Internet]. 2020 [cited 2024 Jun 14];72(6):939–947. Available from: https://pubmed.ncbi.nlm.nih.gov/31588781/31588781 10.1080/01635581.2019.1670852

[145] Bredahl EC, Hydock DS. Creatine supplementation and doxorubicin-induced skeletal muscle dysfunction: an ex vivo investigation. Nutr Cancer [Internet]. 2017 [cited 2024 Jun 14];69(4):607–615. Available from: https://pubmed.ncbi.nlm.nih.gov/28323480/28323480 10.1080/01635581.2017.1295089

[146] Costa Godinho LRL, Cella PS, Guimarães TAS, et al. Creatine supplementation potentiates exercise protective effects against doxorubicin-induced hepatotoxicity in mice. Antioxidants (Basel) [Internet]. 2023 [cited 2024 Jun 14];12(4):823. Available from: https://pubmed.ncbi.nlm.nih.gov/37107198/37107198 10.3390/antiox12040823 PMC10135080

[147] Torok ZA, Busekrus RB, Hydock DS. Effects of creatine supplementation on muscle fatigue in rats receiving doxorubicin treatment. Nutr Cancer [Internet]. 2020 [cited 2024 Jun 14];72(2):252–259. Available from: https://pubmed.ncbi.nlm.nih.gov/31184509/31184509 10.1080/01635581.2019.1623900

[148] Law D, Magrini MA, Siedlik JA, et al. Creatine and resistance training: a combined approach to attenuate doxorubicin-induced cardiotoxicity. Nutrients [Internet]. 2023 [cited 2024 Jun 14];15(18):4048. Available from: https://pubmed.ncbi.nlm.nih.gov/37764831/37764831 10.3390/nu15184048 PMC10536171

[149] Bourgeois JM, Nagel K, Pearce E, et al. Creatine monohydrate attenuates body fat accumulation in children with acute lymphoblastic leukemia during maintenance chemotherapy. Pediatr Blood Cancer [Internet]. 2008 [cited 2022 Apr 5];51(2):183–187. Available from: https://pubmed.ncbi.nlm.nih.gov/18421708/18421708 10.1002/pbc.21571

[150] Nawata CM, Pannabecker TL. Mammalian urine concentration: a review of renal medullary architecture and membrane transporters. J Comp Physiol B [Internet]. 2018 [cited 2024 Jun 14];188(6):899–918. Available from: https://pubmed.ncbi.nlm.nih.gov/29797052/29797052 10.1007/s00360-018-1164-3 PMC6186196

[151] Kreider RB, Melton C, Rasmussen CJ, et al. Long-term creatine supplementation does not significantly affect clinical markers of health in athletes. Mol Cell Biochem. 2003;244(1/2):95–104. doi: 10.1023/A:1022469320296 12701816

[152] Riesberg LA, Weed SA, McDonald TL, et al. Beyond muscles: the untapped potential of creatine. Int Immunopharmacol [Internet]. 2016 [cited 2021 Oct 26];37:31. Available from:/pmc/articles/PMC4915971/26778152 10.1016/j.intimp.2015.12.034 PMC4915971

[153] Brewster LM, Mairuhu G, Bindraban NR, et al. Creatine kinase activity is associated with blood pressure. Circulation [Internet]. 2006 [cited 2024 Jun 14];114(19):2034–2039. Available from: https://pubmed.ncbi.nlm.nih.gov/17075013/17075013 10.1161/CIRCULATIONAHA.105.584490

[154] Armentano M, Brenner A, Hedman T, et al. The effect and safety of short-term creatine supplementation on performance of push-ups. Mil Med [Internet]. 2007 [cited 2021 Oct 6];172(3):312–317. Available from: https://pubmed.ncbi.nlm.nih.gov/17436778/17436778 10.7205/milmed.172.3.312

[155] Mihic S, MacDonald J, McKenzie S, et al. Acute creatine loading increases fat-free mass, but does not affect blood pressure, plasma creatinine, or CK activity in men and women. Med Sci Sports Exerc [Internet]. 2000 [cited 2021 Oct 4];32(2):291–296. Available from: https://pubmed.ncbi.nlm.nih.gov/10694109/10694109 10.1097/00005768-200002000-00007

[156] Murphy AJ, Watsford ML, Coutts AJ, et al. Effects of creatine supplementation on aerobic power and cardiovascular structure and function. J Sci Med Sport. 2005;8(3):305–313. doi: 10.1016/S1440-2440(05)80041-6 16248471

[157] Horjus DL, Oudman I, van Montfrans GA, et al. Creatine and creatine analogues in hypertension and cardiovascular disease. Cochrane Database Syst Rev [Internet]. 2011 [cited 2024 Jun 14]. Available from: https://pubmed.ncbi.nlm.nih.gov/22071819/10.1002/14651858.CD005184.pub2 PMC6823205 22071819

[158] Chilibeck PD, Candow DG, Gordon JJ, et al. A 2-year randomized controlled trial on creatine supplementation during exercise for postmenopausal bone health. Med Sci Sport Exerc. 2023. doi: 10.1249/MSS.0000000000003202 PMC10487398 37144634

[159] Alves CR, Murai IH, Ramona P, et al. No effect of creatine supplementation on oxidative stress and cardiovascular parameters in spontaneously hypertensive rats. J Int Soc Sport Nutr 2012 91 [Internet]. 2012 [cited 2021 Oct 6];9(1):1–4. Available from: https://jissn.biomedcentral.com/articles/10.1186/1550-2783-9-13 10.1186/1550-2783-9-13 PMC3342894 22480293

[160] Sanchez-Gonzalez MA, Wieder R, Kim J-S, et al. Creatine supplementation attenuates hemodynamic and arterial stiffness responses following an acute bout of isokinetic exercise. Eur J Appl Physiol [Internet]. 2011 [cited 2021 Oct 6];111(9):1965–1971. Available from: https://link.springer.com/article/10.1007/s00421-011-1832-4 21249385 10.1007/s00421-011-1832-4

[161] Fakih H, MacLusky N, DeCherney A, et al. Enhancement of human sperm motility and velocity in vitro: effects of calcium and creatine phosphate. Fertil Steril [Internet]. 1986 [cited 2024 Jun 14];46(5):938–944. Available from: https://pubmed.ncbi.nlm.nih.gov/3781011/3781011 10.1016/s0015-0282(16)49839-0

[162] Dickinson H, Ellery S, Ireland Z, et al. Creatine supplementation during pregnancy: summary of experimental studies suggesting a treatment to improve fetal and neonatal morbidity and reduce mortality in high-risk human pregnancy. BMC Pregnancy Childbirth [Internet]. 2014 [cited 2024 Jun 14];14(1). Available from: https://pubmed.ncbi.nlm.nih.gov/24766646/10.1186/1471-2393-14-150 PMC4007139 24766646

[163] Herring CM, Bazer FW, Johnson GA, et al. Impacts of maternal dietary protein intake on fetal survival, growth, and development. Exp Biol Med (Maywood) [Internet]. 2018 [cited 2024 Jun 14];243(6):525–533. Available from: https://pubmed.ncbi.nlm.nih.gov/29466875/29466875 10.1177/1535370218758275 PMC5882021

[164] Muccini AM, Tran NT, de GD, et al. Creatine metabolism in female reproduction, pregnancy and newborn health. Nutrients [Internet]. 2021 [cited 2021 Oct 26];13(2):1–25. Available from:/pmc/articles/PMC7912953/10.3390/nu13020490 PMC7912953 33540766

[165] de Guingand Dl, Palmer KR, Callahan DL, et al. Creatine and pregnancy outcomes: a prospective cohort study of creatine metabolism in low-risk pregnant females. Am J Clin Nutr [Internet]. 2024 [cited 2024 Jun 14];119(3):838–849. Available from: https://pubmed.ncbi.nlm.nih.gov/38432717/38432717 10.1016/j.ajcnut.2023.11.006

[166] Clark LC, Thompson H, Beck EI. The excretion of creatine and creatinine during pregnancy. Am J Obstet Gynecol [Internet]. 1951 [cited 2024 Jun 14];62(3):576–583. Available from: https://pubmed.ncbi.nlm.nih.gov/14877920/14877920 10.1016/0002-9378(51)91156-8

[167] Pinto J, Barros AS, Domingues MRM, et al. Following healthy pregnancy by NMR metabolomics of plasma and correlation to urine. J Proteome Res [Internet]. 2015 [cited 2024 Jun 14];14(2):1263–1274. Available from: https://pubmed.ncbi.nlm.nih.gov/25529102/25529102 10.1021/pr5011982

[168] de Guingand Dl, Palmer KR, Callahan DL, et al. The role of creatine in placental energy metabolism at birth: initial insights from the creatine and pregnancy outcomes (CPO) cohort study of low-risk pregnancy. Placenta. 2023;140:e70. doi: 10.1016/j.placenta.2023.07.221

[169] Mihatsch WA, Stahl B, Braun U. The umbilical cord creatine flux and time course of human milk creatine across lactation. Nutrients [Internet]. 2024 [cited 2024 Jun 26];16(3):345. Available from: https://pubmed.ncbi.nlm.nih.gov/38337631/38337631 10.3390/nu16030345 PMC10857059

[170] Korovljev D, Todorovic N, Stajer V, et al. Dietary intake of creatine in children aged 0–24 months. Ann Nutr Metab [Internet]. 2021 [cited 2024 Jun 26];77(3):185–188. Available from: https://pubmed.ncbi.nlm.nih.gov/33906195/33906195 10.1159/000515917

[171] Ostojic SM, Forbes SC, Candow DG. Do pregnant women consume enough creatine? Evidence from NHANES 2011–2018. Ann Nutr Metab [Internet]. 2022 [cited 2024 Jun 14];78(2):114–116. Available from: https://pubmed.ncbi.nlm.nih.gov/34763333/34763333 10.1159/000520818

[172] Ostojic SM, Stea TH, Ellery SJ, et al. Association between dietary intake of creatine and female reproductive health: evidence from NHANES 2017–2020. Food Sci Nutr [Internet]. 2024 [cited 2024 Jun 14]. Available from: https://onlinelibrary.wiley.com/doi/full/10.1002/fsn3.4135 10.1002/fsn3.4135 PMC11266896 39055234

[173] Berry MJ, Schlegel M, Kowalski GM, et al. UNICORN babies: understanding circulating and cerebral creatine levels of the preterm infant. An observational study protocol. Front Physiol [Internet]. 2019 [cited 2024 Jun 26];10:142. Available from:/pmc/articles/PMC6417365/30899224 10.3389/fphys.2019.00142 PMC6417365

[174] Tran NT, Kelly SB, Snow RJ, et al. Assessing creatine supplementation for neuroprotection against perinatal hypoxic-ischaemic encephalopathy: a systematic review of perinatal and adult pre-clinical studies. Cells [Internet]. 2021 [cited 2024 Jun 14];10(11):2902. Available from: https://pubmed.ncbi.nlm.nih.gov/34831126/34831126 10.3390/cells10112902 PMC8616304

[175] Ellery SJ, Kelleher M, Grigsby P, et al. Antenatal prevention of cerebral palsy and childhood disability: is the impossible possible? J Physiol [Internet]. 2018 [cited 2024 Jun 14];596(23):5593–5609. Available from: https://pubmed.ncbi.nlm.nih.gov/29928763/29928763 10.1113/JP275595 PMC6265564

[176] Perlman JM, Tack ED, Martin T, et al. Acute systemic organ injury in term infants after asphyxia. Am J Dis Child [Internet]. 1989 [cited 2024 Jun 14];143(5):617–620. Available from: https://pubmed.ncbi.nlm.nih.gov/2718998/2718998 10.1001/archpedi.1989.02150170119037

[177] de Guingand Dl, Palmer KR, Bilardi JE, et al. Acceptability of dietary or nutritional supplementation in pregnancy (ADONS) – exploring the consumer’s perspective on introducing creatine monohydrate as a pregnancy supplement. Midwifery [Internet]. 2020 [cited 2024 Jun 14];82. Available from 102599. Available from: https://pubmed.ncbi.nlm.nih.gov/31877396/31877396 10.1016/j.midw.2019.102599

[178] Ireland Z, Dickinson H, Snow R, et al. Maternal creatine: does it reach the fetus and improve survival after an acute hypoxic episode in the spiny mouse (acomys cahirinus)? Am J Obstet Gynecol [Internet]. 2008 [cited 2024 Jun 14];198(4):.e431.1–.e431.6. Available from: https://pubmed.ncbi.nlm.nih.gov/18295173/10.1016/j.ajog.2007.10.790 18295173

[179] LaRosa DA, Ellery SJ, Parkington HC, et al. Maternal creatine supplementation during pregnancy prevents long-term changes in diaphragm muscle structure and function after birth asphyxia. PLoS One [Internet]. 2016 [cited 2024 Jun 14];11(3):e0149840. Available from: https://pubmed.ncbi.nlm.nih.gov/26930669/26930669 10.1371/journal.pone.0149840 PMC4773130

[180] Larosa DA, Ellery SJ, Snow RJ, et al. Maternal creatine supplementation during pregnancy prevents acute and long-term deficits in skeletal muscle after birth asphyxia: a study of structure and function of hind limb muscle in the spiny mouse. Pediatr Res [Internet]. 2016 [cited 2024 Jun 14];80(6):852–860. Available from: https://pubmed.ncbi.nlm.nih.gov/27466898/27466898 10.1038/pr.2016.153

[181] Ellery SJ, Larosa DA, Cullen-Mcewen LA, et al. Renal dysfunction in early adulthood following birth asphyxia in male spiny mice, and its amelioration by maternal creatine supplementation during pregnancy. Pediatr Res [Internet]. 2017 [cited 2024 Jun 14];81(4):646–653. Available from: https://pubmed.ncbi.nlm.nih.gov/27997529/27997529 10.1038/pr.2016.268

[182] Ellery SJ, LaRosa DA, Kett MM, et al. Dietary creatine supplementation during pregnancy: a study on the effects of creatine supplementation on creatine homeostasis and renal excretory function in spiny mice. Amino Acids [Internet]. 2016 [cited 2024 Jun 14];48(8):1819–1830. Available from: https://pubmed.ncbi.nlm.nih.gov/26695944/26695944 10.1007/s00726-015-2150-7

[183] Tran NT, Muccini AM, Snow RJ, et al. The physiological effects of creatine supplementation in fetal sheep before, during, and after umbilical cord occlusion and global hypoxia. J Appl Physiol [Internet]. 2021 [cited 2024 Jun 14];131(3):1088–1099. Available from: https://pubmed.ncbi.nlm.nih.gov/34382841/34382841 10.1152/japplphysiol.00092.2021

[184] Tran NT, Kowalski GM, Muccini AM, et al. Creatine supplementation reduces the cerebral oxidative and metabolic stress responses to acute in utero hypoxia in the late-gestation fetal sheep. J Physiol [Internet]. 2022 [cited 2024 Jun 14];600(13):3193–3210. Available from: https://pubmed.ncbi.nlm.nih.gov/35587817/35587817 10.1113/JP282840 PMC9542404

[185] Muccini AM, Tran NT, Hale N, et al. The effects of in utero fetal hypoxia and creatine treatment on mitochondrial function in the late gestation fetal sheep brain. Oxid Med Cell Longev [Internet]. 2022 [cited 2024 Jun 14];2022. Available from 1–19. Available from: https://pubmed.ncbi.nlm.nih.gov/35132347/10.1155/2022/3255296 PMC8817846 35132347

[186] Tran NT, Muccini AM, Hale N, et al. Creatine in the fetal brain: a regional investigation of acute global hypoxia and creatine supplementation in a translational fetal sheep model. Front Cell Neurosci [Internet]. 2023 [cited 2024 Jun 14];17. Available from: https://pubmed.ncbi.nlm.nih.gov/37066075/10.3389/fncel.2023.1154772 PMC10097948 37066075

[187] Sartini S, Lattanzi D, Ambrogini P, et al. Maternal creatine supplementation affects the morpho-functional development of hippocampal neurons in rat offspring. Neuroscience [Internet]. 2016 [cited 2024 Jun 14];312:120–129. Available from: https://pubmed.ncbi.nlm.nih.gov/26592720/26592720 10.1016/j.neuroscience.2015.11.017

[188] Sartini S, Lattanzi D, Di Palma M, et al. Maternal creatine supplementation positively affects male rat hippocampal synaptic plasticity in adult offspring. Nutrients [Internet]. 2019 [cited 2024 Jun 14];11(9):2014. Available from: https://pubmed.ncbi.nlm.nih.gov/31461895/31461895 10.3390/nu11092014 PMC6770830

[189] de GD, Palmer KR, Snow RJ, et al. Risk of adverse outcomes in females taking oral creatine monohydrate: a systematic review and meta-analysis. Nutrients [Internet]. 2020 [cited 2021 Oct 5];12(6):1–26. Available from:/pmc/articles/PMC7353222/10.3390/nu12061780 PMC7353222 32549301

[190] Wax B, Kerksick C, Jagim A, et al. Creatine for exercise and sports performance, with recovery considerations for healthy populations. Nutrients [Internet]. 2021 [cited 2021 Jul 8];13(6):1915. Available from: https://pubmed.ncbi.nlm.nih.gov/34199588/34199588 10.3390/nu13061915 PMC8228369

[191] Kreider RB. Effects of creatine supplementation on performance and training adaptations. Mol Cell Biochem. 2003;244(1/2):89–94. doi: 10.1023/A:1022465203458 12701815

[192] Dawson B, Vladich T, Blanksby B. Effects of 4 weeks of creatine supplementation in junior swimmers on freestyle sprint and swim bench performance. J Strength Cond Res [Internet]. 2002 [cited 2021 Oct 6];16(4):485–490. Available from: https://pubmed.ncbi.nlm.nih.gov/12423175/12423175

[193] Grindstaff P, Kreider R, Bishop R, et al. Effects of creatine supplementation on repetitive sprint performance and body composition in competitive swimmers. Int J Sport Nutr [Internet]. 1997 [cited 2021 Oct 6];7(4):330–348. Available from: https://pubmed.ncbi.nlm.nih.gov/9407259/9407259 10.1123/ijsn.7.4.330

[194] Juhász I, Györe I, Csende Z, et al. Creatine supplementation improves the anaerobic performance of elite junior fin swimmers. Acta Physiol Hung [Internet]. 2009 [cited 2021 Oct 6];96(3):325–336. Available from: https://akjournals.com/view/journals/036/96/3/article-p325.xml 19706374 10.1556/APhysiol.96.2009.3.6

[195] Theodorou A, Cooke C, King R, et al. The effect of longer-term creatine supplementation on elite swimming performance after an acute creatine loading. J Sports Sci [Internet]. 1999 [cited 2021 Oct 6];17:853–859. Available from: https://pubmed.ncbi.nlm.nih.gov/10585165/10585165 10.1080/026404199365416

[196] Theodorou A, Havenetidis K, Zanker C, et al. Effects of acute creatine loading with or without carbohydrate on repeated bouts of maximal swimming in high-performance swimmers. J Strength Cond Res [Internet]. 2005 [cited 2021 Oct 6];19(2):265–269. Available from: https://pubmed.ncbi.nlm.nih.gov/15903360/15903360 10.1519/15314.1

[197] Claudino JG, Mezêncio B, Amaral S, et al. Creatine monohydrate supplementation on lower-limb muscle power in Brazilian elite soccer players. J Int Soc Sports Nutr [Internet]. 2014 [cited 2021 Oct 6];11(1):32. Available from:/pmc/articles/PMC4077550/24991195 10.1186/1550-2783-11-32 PMC4077550

[198] Mohebbi H, Rahnama N, Moghadassi M, et al. Effect of creatine supplementation on sprint and skill performance in young soccer players. Middle East J Sci Res. 2012;12:397–401.

[199] Ostojic SM. Creatine supplementation in young soccer players. Int J Sport Nutr Exerc Metab [Internet]. 2004 [cited 2021 Oct 6];14(1):95–103. Available from: https://journals.humankinetics.com/view/journals/ijsnem/14/1/article-p95.xml 15129933 10.1123/ijsnem.14.1.95

[200] Yáñez-Silva A, Buzzachera CF, Piçarro IDC, et al. Effect of low dose, short-term creatine supplementation on muscle power output in elite youth soccer players. J Int Soc Sports Nutr [Internet]. 2017 [cited 2021 Oct 6];14(1):5. Available from:/pmc/articles/PMC5296953/28190980 10.1186/s12970-017-0162-2 PMC5296953

[201] Ojeda Á H, Jofré-Saldía E, Torres-Banduc M, et al. Effects of a low dose of orally administered creatine monohydrate on post-fatigue muscle power in young soccer players. Nutrients [Internet]. 2024 [cited 2024 Jun 14];16(9):1324. Available from: https://pubmed.ncbi.nlm.nih.gov/38732571/38732571 10.3390/nu16091324 PMC11085131

[202] Jagim AR, Stecker RA, Harty PS, et al. Safety of creatine supplementation in active adolescents and youth: a brief review. Front Nutr. 2018;5:115. doi: 10.3389/fnut.2018.00115 30547033 PMC6279854

[203] Metzger GA, Minneci PM, Gehred A, et al. Creatine supplementation in the pediatric and adolescent athlete– a literature review. J Orthop [Internet]. 2023 [cited 2024 Jun 14];38:73–78. Available from: https://pubmed.ncbi.nlm.nih.gov/37008451/37008451 10.1016/j.jor.2023.03.010 PMC10063391

[204] Juhasz I, Kopkane J, Hajdu P, et al. Creatine supplementation supports the rehabilitation of adolescent fin swimmers in tendon overuse injury cases. J Sports Sci Med. 2018;17:279–288. 29769829 PMC5950745

[205] El Osta R, Almont T, Diligent C, et al. Anabolic steroids abuse and male infertility. Basic Clin Androl [Internet]. 2016 [cited 2024 Jun 14];26(1). Available from: https://pubmed.ncbi.nlm.nih.gov/26855782/10.1186/s12610-016-0029-4 PMC4744441 26855782

[206] Ketheeswaran S, Haahr T, Povlsen B, et al. Protein supplementation intake for bodybuilding and resistance training may impact sperm quality of subfertile men undergoing fertility treatment: a pilot study. Asian J Androl [Internet]. 2019 [cited 2024 Jun 14];21(2):208–211. Available from: https://pubmed.ncbi.nlm.nih.gov/30226218/30226218 10.4103/aja.aja_49_18 PMC6413551

[207] Powers ME, Arnold BL, Weltman AL, et al. Creatine supplementation increases total body water without altering fluid distribution. J Athl Train [Internet]. 2003 [cited 2024 Jun 14];38:44. Available from:/pmc/articles/PMC155510/12937471 PMC155510

[208] Nasrallah F, Hammami MB, Omar S, et al. Semen creatine and creatine kinase activity as an indicator of sperm quality. Clin Lab [Internet]. 2020 [cited 2024 Jun 14];66(9/2020):1751–1757. Available from: https://pubmed.ncbi.nlm.nih.gov/32902220/10.7754/Clin.Lab.2020.191248 32902220

[209] Iyer GS, Krahe R, Goodwin LA, et al. Identification of a testis-expressed creatine transporter gene at 16p11.2 and confirmation of the X-linked locus to Xq28. Genomics [Internet]. 1996 [cited 2024 Jun 14];34(1):143–146. Available from: https://pubmed.ncbi.nlm.nih.gov/8661037/8661037 10.1006/geno.1996.0254

[210] Ostojic SM, Stea TH, Engeset D. Creatine as a promising component of paternal preconception diet. Nutrients [Internet]. 2022 [cited 2024 Jun 14];14(3):586. Available from: https://pubmed.ncbi.nlm.nih.gov/35276945/35276945 10.3390/nu14030586 PMC8839819

[211] Umehara T, Kawai T, Goto M, et al. Creatine enhances the duration of sperm capacitation: a novel factor for improving in vitro fertilization with small numbers of sperm. Hum Reprod [Internet]. 2018 [cited 2024 Jun 14];33(6):1117–1129. Available from: https://pubmed.ncbi.nlm.nih.gov/29635630/29635630 10.1093/humrep/dey081 PMC5972610

[212] Umehara T, Tsujita N, Goto M, et al. Methyl-beta cyclodextrin and creatine work synergistically under hypoxic conditions to improve the fertilization ability of boar ejaculated sperm. Anim Sci J [Internet]. 2020 [cited 2024 Jun 14];91(1). Available from: https://pubmed.ncbi.nlm.nih.gov/33314533/10.1111/asj.13493 33314533

[213] Tapeh RS, Zhandi M, Zaghari M, et al. Effects of guanidinoacetic acid diet supplementation on semen quality and fertility of broiler breeder roosters. Theriogenology [Internet]. 2017 [cited 2024 Jun 14];89:178–182. Available from: https://pubmed.ncbi.nlm.nih.gov/28043349/28043349 10.1016/j.theriogenology.2016.11.012

[214] Talbot SA, Diaz FA, Gutierrez-Castillo EJ, et al. 94 Effect of glycine and creatine on the. Reprod Fertil Dev [Internet]. 2021 [cited 2024 Jun 14];34(2):284. Available from: https://pubmed.ncbi.nlm.nih.gov/35231224/10.1071/RDv34n2Ab94 35231224

[215] Tøttenborg SS, Glazer CH, Hærvig KK, et al. Semen quality among young healthy men taking protein supplements. Fertil Steril [Internet]. 2020 [cited 2024 Jun 14];114(1):89–96. Available from: https://pubmed.ncbi.nlm.nih.gov/32387273/32387273 10.1016/j.fertnstert.2020.02.103

[216] Candow DG, Ostojic SM, Forbes SC, et al. Does one dose of creatine supplementation fit all? Adv Exerc Heal Sci [Internet]. 2024 [cited 2024 Jun 3]. Available from: https://linkinghub.elsevier.com/retrieve/pii/S2950273X24000249

[217] Forbes SC, Cordingley DM, Cornish SM, et al. Effects of creatine supplementation on brain function and health. Nutrients [Internet]. 2022 [cited 2022 Apr 5];14(5):921. Available from: https://pubmed.ncbi.nlm.nih.gov/35267907/35267907 10.3390/nu14050921 PMC8912287

[218] Harris R, Söderlund K, Hultman E. Elevation of creatine in resting and exercised muscle of normal subjects by creatine supplementation. Clin Sci [Internet]. 1992 [cited 2021 Oct 26];83(3):367–374. Available from https://pubmed.ncbi.nlm.nih.gov/1327657/10.1042/cs0830367 1327657

[219] Steenge GR, Lambourne J, Casey A, et al. Stimulatory effect of insulin on creatine accumulation in human skeletal muscle. Am J Physiol - Endocrinol Metab [Internet]. 1998 [cited 2022 Jan 20];275(6):E974–979. Available from: https://journals.physiology.org/doi/abs/10.1152/ajpendo.1998.275.6.E974 10.1152/ajpendo.1998.275.6.E974 9843739

[220] Candow DG, Forbes SC, Ostojic SM, et al. “Heads up” for creatine supplementation and its potential applications for brain health and function. Sports Med [Internet]. 2023 [cited 2023 Jul 19]. Available from: https://pubmed.ncbi.nlm.nih.gov/37368234/10.1007/s40279-023-01870-9 PMC10721691 37368234

[221] Dechent P, Pouwels PJW, Wilken B, et al. Increase of total creatine in human brain after oral supplementation of creatine-monohydrate. Am J Physiol [Internet]. 1999 [cited 2022 Dec 18];277(3):R698–R704. Available from: https://pubmed.ncbi.nlm.nih.gov/10484486/10484486 10.1152/ajpregu.1999.277.3.R698

[222] Gordji-Nejad A, Matusch A, Kleedörfer S, et al. Single dose creatine improves cognitive performance and induces changes in cerebral high energy phosphates during sleep deprivation. Sci Rep [Internet]. 2024 [cited 2024 Mar 27];14(1). Available from: https://pubmed.ncbi.nlm.nih.gov/38418482/10.1038/s41598-024-54249-9 PMC10902318 38418482

[223] Pan JW, Takahashi K. Cerebral energetic effects of creatine supplementation in humans. Am J Physiol Regul Integr Comp Physiol [Internet]. 2007 [cited 2021 Oct 29];292(4):R1745. Available from:/pmc/articles/PMC3570026/17185404 10.1152/ajpregu.00717.2006 PMC3570026

[224] Slankamenac J, Ranisavljev M, Todorovic N, et al. Effects of six-month creatine supplementation on patient- and clinician-reported outcomes, and tissue creatine levels in patients with post-COVID-19 fatigue syndrome. Food Sci Nutr [Internet]. 2023 [cited 2024 Mar 19];11(11):6899–6906. Available from: https://pubmed.ncbi.nlm.nih.gov/37970399/37970399 10.1002/fsn3.3597 PMC10630839

[225] Solis M, Artioli G, Otaduy M, et al. Effect of age, diet, and tissue type on PCr response to creatine supplementation. J Appl Physiol [Internet]. 2017 [cited 2021 Oct 29];123(2):407–414. Available from: https://pubmed.ncbi.nlm.nih.gov/28572496/28572496 10.1152/japplphysiol.00248.2017

[226] Dolan E, Gualano B, Rawson ES. Beyond muscle: the effects of creatine supplementation on brain creatine, cognitive processing, and traumatic brain injury. Eur J Sport Sci. 2019;19(1):1–14. doi: 10.1080/17461391.2018.1500644 30086660

[227] Moriarty T, Bourbeau K, Dorman K, et al. Dose–response of creatine supplementation on cognitive function in healthy young adults. Brain Sci [Internet]. 2023 [cited 2024 May 10];13(9):1276. Available from: https://pubmed.ncbi.nlm.nih.gov/37759877/37759877 10.3390/brainsci13091276 PMC10526554

[228] Braissant O, Agat HH. GAMT and SLC6A8 distribution in the central nervous system, in relation to creatine deficiency syndromes: a review. J Inherit Metab Dis [Internet]. 2008 [cited 2022 Dec 18];31(2):230–239. Available from: https://pubmed.ncbi.nlm.nih.gov/18392746/18392746 10.1007/s10545-008-0826-9

[229] Kondo DG, Sung YH, Hellem TL, et al. Open-label adjunctive creatine for female adolescents with ssri-resistant major depressive disorder: a 31-phosphorus magnetic resonance spectroscopy study. J Affect Disord. 2011;135(1–3):354–361. doi: 10.1016/j.jad.2011.07.010 21831448 PMC4641570

[230] McMorris T, Harris RC, Swain J, et al. Effect of creatine supplementation and sleep deprivation, with mild exercise, on cognitive and psychomotor performance, mood state, and plasma concentrations of catecholamines and cortisol. Psychopharmacology (Berl) [Internet]. 2006 [cited 2021 Oct 29];185(1):93–103. Available from: https://link.springer.com/article/10.1007/s00213-005-0269-z 16416332 10.1007/s00213-005-0269-z

[231] McMorris T, Harris RC, Howard AN, et al. Creatine supplementation, sleep deprivation, cortisol, melatonin and behavior. Physiol Behav [Internet]. 2007 [cited 2022 Dec 17];90(1):21–28. Available from: https://pubmed.ncbi.nlm.nih.gov/17046034/17046034 10.1016/j.physbeh.2006.08.024

[232] Cook CJ, Crewther BT, Kilduff LP, et al. Skill execution and sleep deprivation: effects of acute caffeine or creatine supplementation - a randomized placebo-controlled trial. J Int Soc Sports Nutr [Internet]. 2011 [cited 2022 Dec 18];8(1). Available from: https://pubmed.ncbi.nlm.nih.gov/21324203/10.1186/1550-2783-8-2 PMC3049131 21324203

[233] Rawson ES, Lieberman HR, Walsh TM, et al. Creatine supplementation does not improve cognitive function in young adults. Physiol Behav [Internet]. 2008 [cited 2022 Dec 17];95(1–2):130–134. Available from: https://pubmed.ncbi.nlm.nih.gov/18579168/18579168 10.1016/j.physbeh.2008.05.009

[234] Roschel H, Gualano B, Ostojic SM, et al. Creatine supplementation and brain health. Nutrients [Internet]. 2021 [cited 2021 Oct 26];13(2):1–10. Available from:/pmc/articles/PMC7916590/10.3390/nu13020586 PMC7916590 33578876

[235] Signoretti S, Di Pietro V, Vagnozzi R, et al. Transient alterations of creatine, creatine phosphate, N-acetylaspartate and high-energy phosphates after mild traumatic brain injury in the rat. Mol Cell Biochem [Internet]. 2010 [cited 2022 Jan 21];333(1–2):269–277. Available from: https://pubmed.ncbi.nlm.nih.gov/19688182/19688182 10.1007/s11010-009-0228-9

[236] Vagnozzi R, Signoretti S, Floris R, et al. Decrease in N-acetylaspartate following concussion may be coupled to decrease in creatine. J Head Trauma Rehabil [Internet]. 2013 [cited 2022 Jan 21];28(4):284–292. Available from: https://pubmed.ncbi.nlm.nih.gov/23249772/23249772 10.1097/HTR.0b013e3182795045

[237] Ainsley Dean PJ, Arikan G, Opitz B, et al. Potential for use of creatine supplementation following mild traumatic brain injury. Concussion (Lond, Engl) [Internet]. 2017 [cited 2022 Jan 21];2(2):CNC34. Available from: https://pubmed.ncbi.nlm.nih.gov/30202575/10.2217/cnc-2016-0016 PMC6094347 30202575

[238] Sullivan P, Geiger J, Mattson M, et al. Dietary supplement creatine protects against traumatic brain injury. Ann Neurol [Internet]. 2000 [cited 2021 Oct 25];48(5):723–729. Available from: https://pubmed.ncbi.nlm.nih.gov/11079535/11079535

[239] Sakellaris G, Kotsiou M, Tamiolaki M, et al. Prevention of complications related to traumatic brain injury in children and adolescents with creatine administration: an open label randomized pilot study. J Trauma [Internet]. 2006 [cited 2022 Dec 18];61(2):322–329. Available from: https://pubmed.ncbi.nlm.nih.gov/16917445/16917445 10.1097/01.ta.0000230269.46108.d5

[240] Sakellaris G, Nasis G, Kotsiou M, et al. Prevention of traumatic headache, dizziness and fatigue with creatine administration. A pilot study. Acta Paediatr [Internet]. 2008 [cited 2021 Oct 6];97(1):31–34. Available from:/pmc/articles/PMC2583396/10.1111/j.1651-2227.2007.00529.x PMC2583396 18053002

[241] Sakellaris G, Partalis N, Nasis G, et al. Outcome of traumatic dysarthria and lingual problems of understanding with creatine administration. An open label randomized Pilot study. J Trauma Treat [Internet]. 2012 [cited 2022 Jan 21];1(3):120. Available from: https://www.hilarispublisher.com/open-access/outcome-of-traumatic-dysarthria-and-lingual-problems-of-understanding-with-creatine-administration.-an-open-label-randomized-pilot-study-2167-1222.1000120.pdf

[242] Joyce JM, La PL, Walker R, et al. Magnetic resonance spectroscopy of traumatic brain injury and subconcussive hits: a systematic review and meta–analysis. J Neurotrauma [Internet]. 2022 [cited 2024 Jun 17];39(21–22):1455–1476. Available from: https://pubmed.ncbi.nlm.nih.gov/35838132/35838132 10.1089/neu.2022.0125 PMC9689773

[243] Alosco ML, Tripodis Y, Rowland B, et al. A magnetic resonance spectroscopy investigation in symptomatic former NFL players. Brain Imaging Behav [Internet]. 2020 [cited 2023 Apr 28];14(5):1419–1429. Available from: https://link.springer.com/article/10.1007/s11682-019-00060-4 30848432 10.1007/s11682-019-00060-4 PMC6994233

[244] Bødker RL, Marcussen M. Pilot study protocol of a randomized controlled trial for the potential effects of creatine monohydrate on persistent post-concussive symptoms. Front Neurol [Internet]. 2023 [cited 2024 Jun 17];14. Available from: https://pubmed.ncbi.nlm.nih.gov/37475743/10.3389/fneur.2023.1209548 PMC10354866 37475743

[245] Ostojic SM, Grasaas E, Baltic S, et al. Dietary creatine is associated with lower serum neurofilament light chain levels. Appl Physiol Nutr Metab [Internet]. 2024 [cited 2024 Jun 26]. Available from: https://pubmed.ncbi.nlm.nih.gov/38780027/10.1139/apnm-2024-0064 38780027

